# Context matters: the landscape matrix determines the population genetic structure of temperate forest herbs across Europe

**DOI:** 10.1007/s10980-021-01376-7

**Published:** 2021-12-01

**Authors:** Tobias Naaf, Jannis Till Feigs, Siyu Huang, Jörg Brunet, Sara A. O. Cousins, Guillaume Decocq, Pieter De Frenne, Martin Diekmann, Sanne Govaert, Per-Ola Hedwall, Jonathan Lenoir, Jaan Liira, Camille Meeussen, Jan Plue, Pieter Vangansbeke, Thomas Vanneste, Kris Verheyen, Stephanie I. J. Holzhauer, Katja Kramp

**Affiliations:** 1 Leibniz Centre for Agricultural Landscape Research (ZALF), Eberswalder Strasse 84, 15374 Müncheberg, Germany; 2 Southern Swedish Forest Research Centre, Swedish University of Agricultural Sciences, Box 190, 234 22 Lomma, Sweden; 3 Landscapes, Environment and Geomatics, Department of Physical Geography, Stockholm University, 10691 Stockholm, Sweden; 4 Ecologie et Dynamique des Systèmes Anthropisés (EDYSAN, UMR 7058 CNRS), Université de Picardie Jules Verne, 1 Rue des Louvels, 80037 Amiens, France; 5 Forest and Nature Lab, Department of Environment, Faculty of Bioscience Engineering, Ghent University, Geraardsbergsesteenweg 267, 9090 Gontrode-Melle, Belgium; 6 Vegetation Ecology and Conservation Biology, Institute of Ecology, FB2, University of Bremen, Leobener Str., 28359 Bremen, Germany; 7 Institute of Ecology and Earth Sciences, University of Tartu, Lai 40, 51005 Tartu, Estonia; 8 IVL Swedish Environmental Institute, Valhallavägen 81, 10031 Stockholm, Sweden

**Keywords:** Arable crops, Dispersal vectors, Functional connectivity, Genetic differentiation, Genetic diversity, Linear landscape elements

## Abstract

**Context:**

Plant populations in agricultural landscapes are mostly fragmented and their functional connectivity often depends on seed and pollen dispersal by animals. However, little is known about how the interactions of seed and pollen dispersers with the agricultural matrix translate into gene flow among plant populations.

**Objectives:**

We aimed to identify effects of the landscape structure on the genetic diversity within, and the genetic differentiation among, spatially isolated populations of three temperate forest herbs. We asked, whether different arable crops have different effects, and whether the orientation of linear landscape elements relative to the gene dispersal direction matters.

**Methods:**

We analysed the species’ population genetic structures in seven agricultural landscapes across temperate Europe using microsatellite markers. These were modelled as a function of landscape composition and configuration, which we quantified in buffer zones around, and in rectangular landscape strips between, plant populations.

**Results:**

Landscape effects were diverse and often contrasting between species, reflecting their association with different pollen- or seed dispersal vectors. Differentiating crop types rather than lumping them together yielded higher proportions of explained variation. Some linear landscape elements had both a channelling and hampering effect on gene flow, depending on their orientation.

**Conclusions:**

Landscape structure is a more important determinant of the species’ population genetic structure than habitat loss and fragmentation per se. Landscape planning with the aim to enhance the functional connectivity among spatially isolated plant populations should consider that even species of the same ecological guild might show distinct responses to the landscape structure.

**Supplementary Information:**

The online version contains supplementary material available at 10.1007/s10980-021-01376-7.

## Introduction

In many regions on earth, the progressive occupation of land by humans for settlements and agriculture has forced wildlife and wildflowers to live in small remaining fragments of once contiguous natural habitats (Kennedy et al. 2019). Surviving in a system of habitat fragments only succeeds if local populations are functionally connected through the regular exchange of individuals or diaspores. Plants with a limited dispersal potential, low seed production, a transient seed bank and a high age of first sexual reproduction appear poorly equipped to establish such regional population dynamics (Eriksson 1996). Typical temperate forest herbs belong to this group of plants (Whigham 2004) as they evolved within landscapes that used to be covered by forest to a much greater extent (Honnay et al. 2005). Numerous population genetic studies revealed that the functional connectivity among temperate forest herb populations may be strongly reduced in landscapes with a high degree of forest fragmentation. For instance, small, spatially isolated populations often exhibit a reduced allelic richness (Vellend 2004; Jacquemyn et al. 2006; Vandepitte et al. 2007; Kolb and Durka 2013; Naaf et al. 2021) and are strongly genetically differentiated from each other (Jacquemyn et al. 2006; Schmidt et al. 2009; Gentili et al. 2018). However, local populations may maintain a high level of genetic diversity (Culley et al. 2007; Toma et al. 2015) and a low level of genetic differentiation among them (Van Rossum et al. 2002; Tomimatsu and Ohara 2003; Jacquemyn et al. 2009) if they are functionally connected by steady gene flow.

Gene flow in plants depends on abiotic or biotic vectors that transport seeds or pollen between populations. For many plants, these vectors are animals that actively cross the landscape matrix, i.e. the non-habitat part of the landscape (Murphy and Lovett-Doust 2004). We can therefore assume that the structure of the matrix has a significant impact on gene flow in plants and thus their susceptibility to detrimental effects of habitat loss and fragmentation. Nevertheless, population genetic studies in general (Holderegger et al. 2010) and on forest herbs in particular (e.g., Tomimatsu and Ohara 2003; Jacquemyn et al. 2006; Vandepitte et al. 2007; Kolb and Durka 2013; Gentili et al. 2018; but see Westerberg and Saura 1994 and Schmidt et al. 2009) largely ignored the matrix.

Several mechanisms of how the matrix may influence the seed and pollen transport among plant populations are conceivable. First, different land-use types may exhibit different degrees of resistance for seed-dispersing animals or pollinators. In general, the landscape permeability for large mammals, such as deer, wild boar or carnivores, increases with forest cover (Coulon et al. 2004; Herrera et al. 2016), while pollinators seem to prefer open landscapes and transport pollen further, when forest cover is low (Kreyer et al. 2004; Kamm et al. 2010). Many mammals avoid proximity to settlements and roads (Trombulak and Frissell 2000; Bonnot et al. 2013). Grasslands and arable fields are avoided by deer and wild boar after harvest when they provide no shelter and little forage biomass (Thurfjell et al. 2009; Morellet et al. 2011). However, maize fields with their specific phenology and structure provide shelter even during late summer and autumn (Keuling et al. 2009; Tillmann 2011) and thus enhance landscape permeability for mammals at a time, when seeds of many forest herb-layer plants are ripe (Heinken and Raudnitschka 2002).

Second, land-use types serving as forage or nesting habitat may affect animals’ abundance and behaviour. The chance for seeds to be dispersed by birds is directly linked to bird abundance (Garcia et al. 2010) and the abundance of woodland birds in agricultural landscapes increases with increasing forest cover (Heikkinen et al. 2004; Radford and Bennett 2007). Mass-flowering crops such as oilseed rape have been shown to enhance the abundance of bumblebee workers (Westphal et al. 2003) and solitary bees (Holzschuh et al. 2013) at the landscape scale. However, whether such an attractive resource pulse results in spill-over to semi-natural habitats and enhanced pollination service to wild plants (Kovacs-Hostyanszki et al. 2013; Ekroos et al. 2015) or a dilution of pollinators and reduced pollination service (Holzschuh et al. 2011, 2016; Riedinger et al. 2015; Proesmans et al. 2019a, b) needs further investigation. More continuous floral resources in semi-natural habitats such as grasslands or hedgerows may sustain a high pollinator richness and abundance in the long term (Kovacs-Hostyanszki et al. 2013; Riedinger et al. 2015; Bartual et al. 2019). The proximity to such semi-natural habitats has been found to enhance the seed-set of insect-pollinated wild plants in agricultural landscapes (Cussans et al. 2010; Jakobsson and Ågren 2014; Chateil and Porcher 2015; Lindgren et al. 2018). Apart from floral resources, pollinators may depend on further food resources. Larva of aphidophagous hoverflies, for instance, whose preferred adult habitat might be deciduous forest, find their prey mostly in arable fields (Meyer et al. 2009). They can profit from high densities of aphids in cereals as well as in oilseed rape (Haenke et al. 2014).

Third, linear landscape elements influence the movement behaviour of seed and pollen dispersal vectors. Roe deer and wild boar, for instance, move preferably along edges such as hedgerows, forest edges and ditches (Saïd and Servanty 2005; Thurfjell et al. 2009; Morellet et al. 2011). Bumblebees and honey bees fly preferably along hedgerows or other linear landscape elements (Cranmer et al. 2012; Collett and Graham 2015). However, hedgerows may also act as relative barriers to pollinator movement when their orientation crosses the flight direction (Wratten et al. 2003; Klaus et al. 2015). Busy roads may act as barrier to the movement of large mammals (Trombulak and Frissell 2000; Breyne et al. 2014), and may also restrict bumblebee or hoverfly movement (Lövei et al. 1998; Bhattacharya et al. 2003; Fitch and Vaidya 2021).

Although it is evident that the agricultural landscape matrix influences the behaviour of pollen- and seed dispersal vectors, it is unclear how this translates into seed and pollen dispersal rates, and thus functional connectivity, among forest herb populations. Here, we therefore quantified the landscape composition and configuration in terms of both area-based land-use types and linear landscape elements in seven agricultural landscapes across north-western Europe to study their effects on the population genetic structure of three common temperate forest herb species: *Anemone nemorosa* L., *Oxalis acetosella* L. and *Polygonatum multiflorum* (L.) All. All three species are typical, slow-colonizing forest specialists, but differ in their reproduction strategy and associated pollen and seed dispersal vectors. Therefore, we expect them to respond differently to the landscape structure (Table 1). In our understanding, the landscape-scale population genetic structure comprises both the genetic diversity within and the genetic differentiation among local populations. We use it here as an indirect measure of functional connectivity among local plant populations (Aavik et al. 2014) and tested the following main hypotheses:

- *H1* Landscape effects on the population genetic structure differ among the three forest herbs because of their association with different pollen and/or seed dispersal vectors.
- *H2* Different arable crops (oilseed rape, maize, other cereals) have different effects on the forest herbs’ population genetic structure due to their differential effect on the associated pollen and/or seed dispersal vectors.
- *H3* Linear landscape elements may have a channelling or impeding effect on gene flow depending on their orientation in relation to gene dispersal pathways.

**Table 1:** Landscape metrics used to study the effects of the landscape composition and configuration on the population genetic structure of the temperate forest herbs *Anemone nemorosa*, *Oxalis* *acetosella*, and *Polygonatum multiflorum*

Landscape metric	Definition (min, median, max)	Expected effect on gene flow among populations^a^			
	Species:	*Anemone nemorosa* *Oxalis acetosella*	*Oxalis acetosella*	*Polygonatum multiflorum*	*Polygonatum multiflorum*
	Vector:	Hoverflies, bees	Wild boar, deer	Bumblebees	Birds, mammals
Area-based metrics					
	Percent cover of …				
FOREST	Deciduous forest (2.5; 8; 23.6)	**−** ^40, 46, 81, 84^, **0** ^51, 86^, + ^30, 50, 54, 59, 60, 70^	** + **^55, 56^	**−** ^22, 40, 46, 81, 84^, **0** ^51^	** + **^5, 9, 20, 29, 31, 34, 38, 63, 67, 78^
GRASS	Grassland in general (2.9; 18.7; 78.9)	**0 + **^3, 11, 39, 45, 66, 72, 73^	**0 + **^2, 71^	**0 + **^22, 37, 80^	**0 + **^5, 23, 52, 63^
SEMNATGRASS	Semi−natural grassland (0; 1.3; 7.3)	** + **^3, 11, 39, 45, 66, 72, 73^	**0 + **^2, 71^	**−** ^64^, + ^11, 17, 22, 61, 80^	** + **^5, 23, 52, 63^
SEMNATVEG	Other semi-natural vegetation (0; 0.7; 8.9)	** + **^3, 11, 39, 66, 72^	**0**	**−** ^64^, + ^11, 17, 61^	** + **^23^
ARABLE	Arable land in general (10.6; 58.8; 81.9)	**−** ^27^,**−+ **^25, 50, 74^	** + **^2, 58, 71^	**−** ^27^	**−** ^5, 29, 63, 67, 68^
RAPE	Arable land cultivated with oilseed rape (0; 4; 17.3)	**−** ^35, 36, 53, 79^,−**+ **^44^, + ^7, 25, 65^	**0**	**−** ^17, 35, 36^, **0** ^22^,**−+ **^44, 83^, + ^26, 33, 43, 82^	**−** ^5, 29, 63, 67, 68^
MAIZE	Arable land cultivated with maize (0; 8.1; 43.4)	**−** ^27^	** + **^2, 8, 32, 41, 71, 76^	**−** ^27^	**−** ^5, 29, 63, 67, 68^
CEREAL	Arable land cultivated with other cereals (2; 21.4; 52.4)	**− + **^25, 50, 74^	** + **^2, 58, 71^	**0**	**−** ^5, 29, 63, 67, 68^
ORCHARD	Traditional grassland orchards (0; 0.1; 3)	**−** ^35^, + ^3, 11, 39, 66, 72^	**0**	**−** ^61^	**0**
SETTLE	Settlement area (0; 4.3; 19)	**0**	**−** ^8, 13^	**−** ^57^,**−+ **^15, 22^	**−** ^5, 13, 67^
+ Conditioning variable: PROPGREEN = Prop. of green urban areas					
Linear landscape elements					
	Relative length [m ha^−1^] of …				
LWOOD	Hedgerows and tree lines (0.4; 27.3; 135.3)	**−** ^85^,**−+ **^12, 42, 45^, + ^1, 16, 21, 25, 59, 70^	**− + **^69, 75^	**− + **^12, 14, 21, 42^	**0 + **^18, 24, 47^, + ^4, 5, 20, 34, 38^
+ Conditioning variable: O:P_LWOOD = Orthogonal-to-parallel ratio					
LWATER	Water courses (incl. draining ditches) (0.9; 11.8; 37.3)	**− + **^12^, + ^1^	**− + **^69, 75^	**− + **^12^	**0**
+ Conditioning variable: O:P_LWATER = Orthogonal-to-parallel ratio					
LFRINGE	Broad herbaceous fringes (0; 3.3; 21.3)	**− + **^12^, ** + **^3, 11, 39, 45, 66, 72, 73^	**− + **^69, 75^	**− + **^12, 43^, ** + **^11, 17, 22, 37, 61^	**0 + **^5, 52, 63^
+ Conditioning variable: O:P_LFRINGE = Orthogonal-to-parallel ratio					
LROAD	Roads (0.7; 15.6; 36.2)	**−** ^48^,−**+ **^12^	**− + **^10, 13, 69, 75, 77^	**− + **^6, 12^	**−** ^5, 13, 19^
+ Conditioning variable: O:P_LROAD = Orthogonal-to-parallel ratio					
Index metrics					
SHANNON	Diversity of land−use types (0.9; 1.7; 2.1)	** + **^11, 72^	**0**	** + **^11, 72^	** + **^29, 67^
EDGEDEN	Land−use parcel edge density [m ha^−1^] (55; 131; 294)	**− + **^49^, + ^28, 72^	**−** ^62, 69^	** + **^72^	**−** ^69^, + ^67, 72^

For each landscape metric, the columns provide the definition and the expected effects on gene flow based on available literature (references indicated by superscripts are provided in Table S5.1). The minimum, median and maximum value of each landscape metric across all populations for a 1000 m buffer zone is given in parentheses. The potential mechanisms via pollen- or seed-dispersal vectors behind expected effects are detailed in Table S5.2

^a^Effect codes:−negative, + positive, −+ negative or positive depending on conditions, 0 no effect, 0 + no or positive effect depending on conditions

## Material and methods

### Study species

The three studied forest herbs were selected for being typical, common slow-colonizing forest specialists (Verheyen et al. 2003; Schmidt et al. 2014). They all exhibit strong clonal growth, but also regular seedling recruitment (Holderegger et al. 1998; Berg 2002; Kosiński 2012). They flower in spring and are pollinated by insects (Klotz et al. 2002). However, they differ in their reproduction strategy. *Oxalis acetosella* has been found to produce most of its seeds from cleistogamous flowers (Berg and Redbo-Torstensson 1998), thus it is less dependent on insect pollinators for sexual reproduction. According to our own research, however, *O. acetosella* is mainly outcrossing (Naaf et al. 2021). *Anemone nemorosa* and *Polygonatum multiflorum* are mainly and strictly outcrossing, respectively (Müller et al. 2000; Kosiński 2012). *Oxalis acetosella* and *A. nemorosa* are pollinated by a wide range of pollinators, including hoverflies, wild bees and honey bees (Shirreffs 1985; Redbo-Torstensson and Berg 1995; Stehlik and Holderegger 2000; Naaf et al. 2021). In contrast, *P. multiflorum* is mostly pollinated by long-tongued bumblebees (Kosiński 2012; Naaf et al. 2021). All three species have a low seed-dispersal potential and are classified as autochorous (Müller-Schneider 1986). In addition, seeds of *A. nemorosa* are dispersed by some short-distance vectors such as ants and slugs (Türke et al. 2012). Seeds of *O. acetosella* were found in the fur of wild boar by several independent studies, though at low quantities (Mrotzek et al. 1999; Heinken and Raudnitschka 2002). The fleshy berries of *P. multiflorum* may suggest endozoochorous dispersal (Müller-Schneider 1986). In fact, however, they are toxic and probably rarely dispersed by birds and mid-sized carnivores such as martens (Ehrlén and Eriksson 1993; Schaumann and Heinken 2002), while short-distance dispersal by small rodents might occur more often (Ehrlén and Eriksson 1993).

### Population genetic structure

**Fig. 1:**
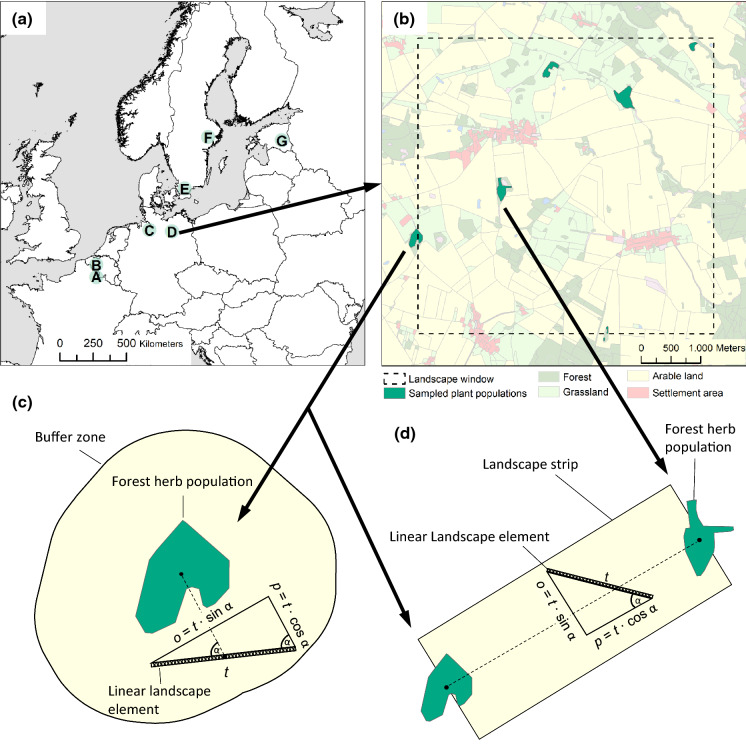
Overview on the study design. The seven 5 × 5 km^2^ landscape windows (**a**) are spread across temperate Europe from North France (A) over Belgium (B), West Germany (C), East Germany (D), and South Sweden (E) up to Central Sweden (F) and Estonia (G). In each agricultural landscape, we surveyed up to six populations of each species, here exemplified for *Polygonatum multiflorum* in East Germany (**b**). See Supp. Inf. 1 for detailed land-use maps of each landscape window, including locations of all sampled populations. The landscape surrounding the populations was analysed at the node level, i.e., in buffer zones of distances between 125 and 2000 m (**c**), and at the link level, i.e. in landscape strips between populations with width-to-length ratios between 1:7 and 2:3 (**d**). For linear landscape elements within buffer zones and landscape strips, we calculated not only the total length (*t*), but also the parallel (*p*) and orthogonal (*o*) length component in relation to the gene dispersal direction

We compiled population genetic data from seven 5 × 5 km^2^ landscape windows spread across north-western Europe from North France, over Belgium, West Germany, East Germany and South Sweden up to Central Sweden and Estonia (Fig. 1a, b). All landscape windows represent typical agricultural landscapes, in which forest fragments are embedded in an agricultural matrix interfused by small settlement areas and roads (see land-use maps in Supp. Inf. S1). We studied up to six forest herb populations from each species in each landscape windows. *Oxalis acetosella* had too few occurrences in Belgium to be included in the analysis of this landscape window. *Polygonatum multiflorum* did not occur in the landscape window of Central Sweden. The final number of surveyed populations was therefore 42, 34 and 36 for *A. nemorosa*, *O. acetosella* and *P. multiflorum*, respectively. Population sizes varied by several orders of magnitude both within and among species ranging from 15 flowering shoots in the smallest *P. multiflorum* population up to > 12*10^6^ flowering shoots in the largest *A. nemorosa* population (Table S2). Geographic distances among populations within landscape windows ranged between 214 and 5518 m and were similarly distributed for each species (Table S2). The population genetic data for these populations comprised four measures of within-population genetic diversity, i.e., allelic richness (*A*_r_), expected heterozygosity (*H*_e_), observed heterozygosity (*H*_o_) and the inbreeding coefficient *F* = 1−*H*_o_/*H*_e_, as well as two measures of among-population genetic differentiation for each pair of populations within landscapes, i.e., *G*’’_ST_ and *D*_PS_ (Table S2). While *G*’’_ST_ is the recommended genetic differentiation measure with microsatellite markers (Meirmans and Hedrick 2011), *D*_PS_ equals 1 minus the proportion of shared alleles and therefore facilitates an intuitive interpretation. The genetic data were based on species-specific sets of nuclear microsatellite markers, which comprised six, nine and six markers with a total number of 102, 61 and 149 alleles for *A. nemorosa*, *O. acetosella* and *P. multiflorum*, respectively (Supp. Inf. S3). While *O. acetosella* and *P. multiflorum* are diploid, *A. nemorosa* was treated as tetraploid (Stehlik and Holderegger 2000). For details on genetic analyses and the calculation of population genetic variables see Naaf et al. (2021), in which we studied the effects of habitat loss and fragmentation per se.

### Landscape metrics

As a basis for our landscape analysis, we created digital land-use maps for all landscape windows based on recent orthophotos and additional, region-specific data (Supp. Inf. S1). Moreover, for all arable fields, we determined the dominance of three different crop types, i.e., oilseed rape, maize and other cereals, over the preceding decade (2008–2017) based on data generated within the European Integrated Administration and Control System (IACS) (European Commission 2020; Supp. Inf. S4). To quantify the composition and configuration of area-based and linear landscape elements, we calculated a set of landscape metrics (Table 1) in (a) buffer zones around each herb population (node-level analysis, Fig. 1c, e.g., Schmidt et al. 2009) and (b) rectangular landscape strips connecting the centres of each pair of herb populations within landscape windows (link-level analysis, Fig. 1d, e.g., Braunisch et al. 2010). Several buffer distances were chosen to reflect range sizes and forage distances of potential seed and pollen dispersal vectors (Table 1): 125 m, 250 m, 500 m, 1000 m and 2000 m. Similarly, we chose several width-to-length ratios for the landscape strips connecting the herb populations to account for the fact that different pollen and seed dispersal vectors have different sight distances for their orientation and thus will move more or less linearly through the landscape: 1:7, 1:5, 1:3, 1:2 and 2:3. For each buffer zone and landscape strip, we calculated the percent cover of different area-based land-use types, the relative length of different linear landscape elements (= total length divided by buffer or strip area, respectively) and two index measures, i.e., the Shannon diversity of land-use types and the density of all land-use patch edges (Table 1). Since the effect that a linear landscape element exerts on gene dispersal might depend on its orientation relative to the movement direction of vectors (orthogonal vs. parallel), we calculated also the orthogonal and parallel length component of each linear landscape element (Fig. 1c, d). In buffer zones, the parallel direction corresponds to the direction from the midpoint of the linear element to the population centre. In landscape strips, the parallel direction corresponds to the connection line between population centres. The orthogonal-to-parallel length ratio was then used as conditioning variable in statistical models (see below). Moreover, the effect of settlement areas on gene dispersal vectors might depend on the relative proportion of sealed or built-up area vs. unsealed green areas, such as gardens. The latter might serve as forage habitat for pollinators, particularly, when many fruit trees or ornamental shrubs can be found there (Cussans et al. 2010; Goulson et al. 2010; Nakamura and Kudo 2019). Therefore, we used also the proportion of green settlement area as conditioning variable in statistical models.

### Data analysis

To study the effects of landscape metrics on genetic diversity (node level) and pairwise genetic differentiation (link level), we used linear mixed-effects models (LMMs) separately for each species with landscape window as random intercept. We fitted these models with the function lme of the R package nlme (Pinheiro et al. 2019). At the link level, we took the correlation among population pairs including a common population into account by defining a correlation structure within the lme function using the function corMLPE (maximum likelihood population-effects models sensu Clarke et al. 2002; Pope 2020). Prior to modelling, all variables were Box-Cox-transformed to increase the symmetry of their distribution and then centred and scaled to yield standardized regression coefficients. We were interested here in marginal effects of the landscape metrics that they exert on genetic variables in addition to those exerted by some basic population genetic determinants, which had been studied earlier (Naaf et al. 2021). At the node level, these basic determinants were population size, i.e., the total number of flowering shoots in a population, and the degree of spatial isolation measured by Hanski’s (1994) incidence function model (see Naaf et al. 2021 for details). At the link level, the basic determinant was edge-to-edge geographical distance. Therefore, all models included population size and isolation or geographic distance, respectively, as fixed effects that could not be removed during model selection. At the link level, we also allowed for interactions between basic determinant and landscape metrics, which was not possible at the node level due to the limited sample size (node level 30–42 vs. link level 78–104; Supp. Inf. S6).

To identify the most important predictors among the large number of landscape metrics and to avoid collinearity, our statistical modelling followed three steps. First, we identified for each landscape metric (Table 1) its most influential buffer distance (node level) or width-to-length ratio (link level) as the one yielding the lowest *AIC*_C_ in LMMs containing only one landscape metric at a time. To account for curvilinear or unimodal relationships these models contained also a quadratic term if this lowered *AIC*_C_. Models for linear landscape elements and settlement area included also the interaction with a corresponding conditioning variable (Table 1). Models at the link level included also an interaction term with geographic distance if this lowered *AIC*_C_. In the following steps, we considered only those most influential landscape metrics that showed a significant effect in the single-metric models at a level of α = 0.15 based on a likelihood ratio test against the reduced model without the landscape metric in question.

Second, we checked for collinearity among the remaining landscape metrics. Any correlations with |*r*|≥ 0.7 were not tolerated. In case of intrinsic collinearity, we used principal components analysis to calculate a principal component of the collinear variables. In case of collinearity among variables that are not obviously ecologically related, we chose the one yielding the highest importance value in the final average model (see below) and excluded the other one (keeping the collinearity in mind for our interpretation). In the special case of percent cover of arable land, we allowed either the percent cover of different crop types (RAPE, MAIZE, CEREAL; Table 1) or the percent cover of arable land in general (ARABLE) to be included to see which version explained a higher proportion of variation in the final average model (see below).

Third, all remaining metrics entered the global model, which was then used for model selection followed by multi-model inference with the R package MuMIn (Barton 2019). In this step, we fitted models using ML estimation for all subsets of predictors allowing for a maximum of four and nine landscape metric terms in models at the node and link level, respectively, given the limited sample size. All models with a Δ*AIC*_C_ < 2 were refit with REML estimation and then subjected to full model averaging (Grueber et al. 2011). For each term in the average model, we calculated an importance value as the sum of the Akaike weights over all component models, in which the term appeared. This importance value ranges between 0 and 1 with a value of 1 indicating that the corresponding term occurs in all component models. Any term with an importance value ≥ 0.5 will be reported and interpreted. To quantify the amount of variation in genetic variables explained by the landscape metrics, we calculated the difference between the marginal *R*^2^ for the average model and the marginal *R*^2^ for the basic model including only the basic population genetic determinants. For the visualization of any important effects (i.e., those with an importance value ≥ 0.5), we used the single best model that included all important terms.

## Results

### Landscape effects at the node level

Out of the 16 landscape metrics considered, 15 were involved in at least one important landscape effect (i.e., with an importance value ≥ 0.5) on a genetic diversity variable (Table 2). The proportion of variation explained uniquely by the landscape effects was mostly larger than the proportion explained by the basic population genetic determinants, population size and spatial isolation (Table 2).

**Table 2:** Summary of landscape effects on allelic richness (*A*_r_), expected (*H*_e_) and observed heterozygosity (*H*_o_) and the inbreeding coefficient (*F*) as resulting from linear mixed-effects models at the node level, separately for (a) *Anemone nemorosa*, (b) *Oxalis acetosella*, and (c) *Polygonatum multiflorum*

Landscape metrics	*A*_r_	*H*_e_	*H*_o_	*F*
(a) *Anemone nemorosa*				
FOREST_2000			\	
ARABLE_2000	/			
RAPE_500				/∩
MAIZE_1000		/		
pcSETTLE_250^a^			/U	
pcSETTLE_500^b^		/		
LROAD_250				/∩
LWOOD_2000	/∩			
LFRINGE_500	\			
Marginal *R*^2^	0.74	0.38	0.60	0.48
Landscape *R*^2^	0.56	0.29	0.58	0.41
% Landscape	76.4	74.9	97.7	84.7
(b) *Oxalis acetosella*				
SEMNATGRASS_1000			U	
SEMNATVEG_500		/∩	/	
MAIZE_250		\∩		
MAIZE_1000				/∩
pcLWOODGRASS_1000^c^				/
LFRINGE_2000				\
LROAD_500	∩			
EDGEDEN_125			\	
Marginal *R*^2^	0.42	0.57	0.42	0.47
Landscape *R*^2^	0.08	0.30	0.19	0.47
% Landscape	19.5	53.0	45.2	99.5
(c) *Polygonatum multiflorum*				
FOREST_2000				/
SEMNATGRASS_250		∩		
pcARABvsGRASS_2000^d^	\	\		
SETTLE_250				\
SETTLE_1000	\	\		
pcSETTLE_1000^e^			\	
LWATER_1000			X	
LWATER_2000	/			
SHANNON_250	/			
LROAD_2000				/
Marginal *R*^2^	0.76	0.38	0.19	0.58
Landscape *R*^2^	0.42	0.24	0.01	0.42
% Landscape	55.9	63.6	4.1	72.3

**Fig. 2:**
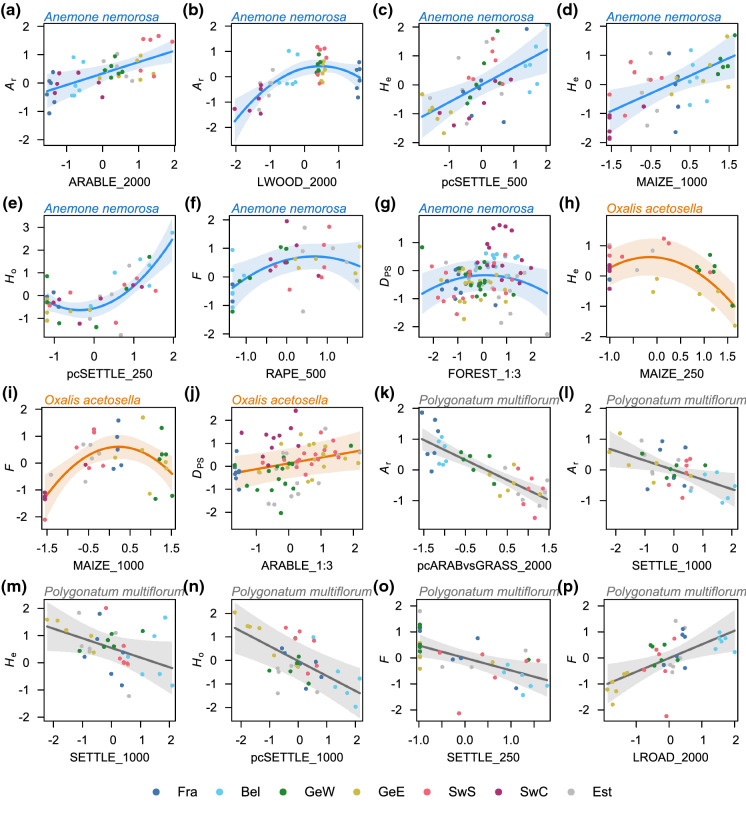
Visualization of landscape effects (cf. Tables 2 and 3) on population genetic variables of *Anemone nemorosa*, *Oxalis acetosella* and *Polygonatum multiflorum*. Shown are those effects, which are directly related to our hypotheses and therefore discussed in the main text. Panels display the partial slopes and residuals as well as the 95% confidence band. All variables are scaled in standard deviation units. Colours of partial residuals represent the different landscape windows: France (Fra), Belgium (Be), West Germany (GeW), East Germany (GeE), South Sweden (SwS), Central Sweden (SwC), and Estonia (Est). Population genetic variables are allelic richness (*A*_r_), expected (*H*_e_) and observed heterozygosity (*H*_o_), inbreeding index (*F*), and genetic differentiation (*D*_PS_). The landscape metrics ARABLE, FOREST, MAIZE, RAPE, and SETTLE refer to the percent cover of arable land, deciduous forest, maize, oilseed rape and settlement area, respectively. LWOOD and LROAD refer to the relative length of hedgerows/tree lines and roads, respectively. pcSETTLE is a principal component reflecting settlement area, road density and edge density (cf. Table 2). pcARABvsGRASS is a principal component reflecting the trade-off between arable land (cereals and oilseed rape) on the one hand and grassland on the other hand (cf. Table 2). Numbers or ratios added to the variable names correspond to the most influential buffer distance in meters or the most influential width-to-length radio of the landscape strips, respectively

**Fig. 3:**
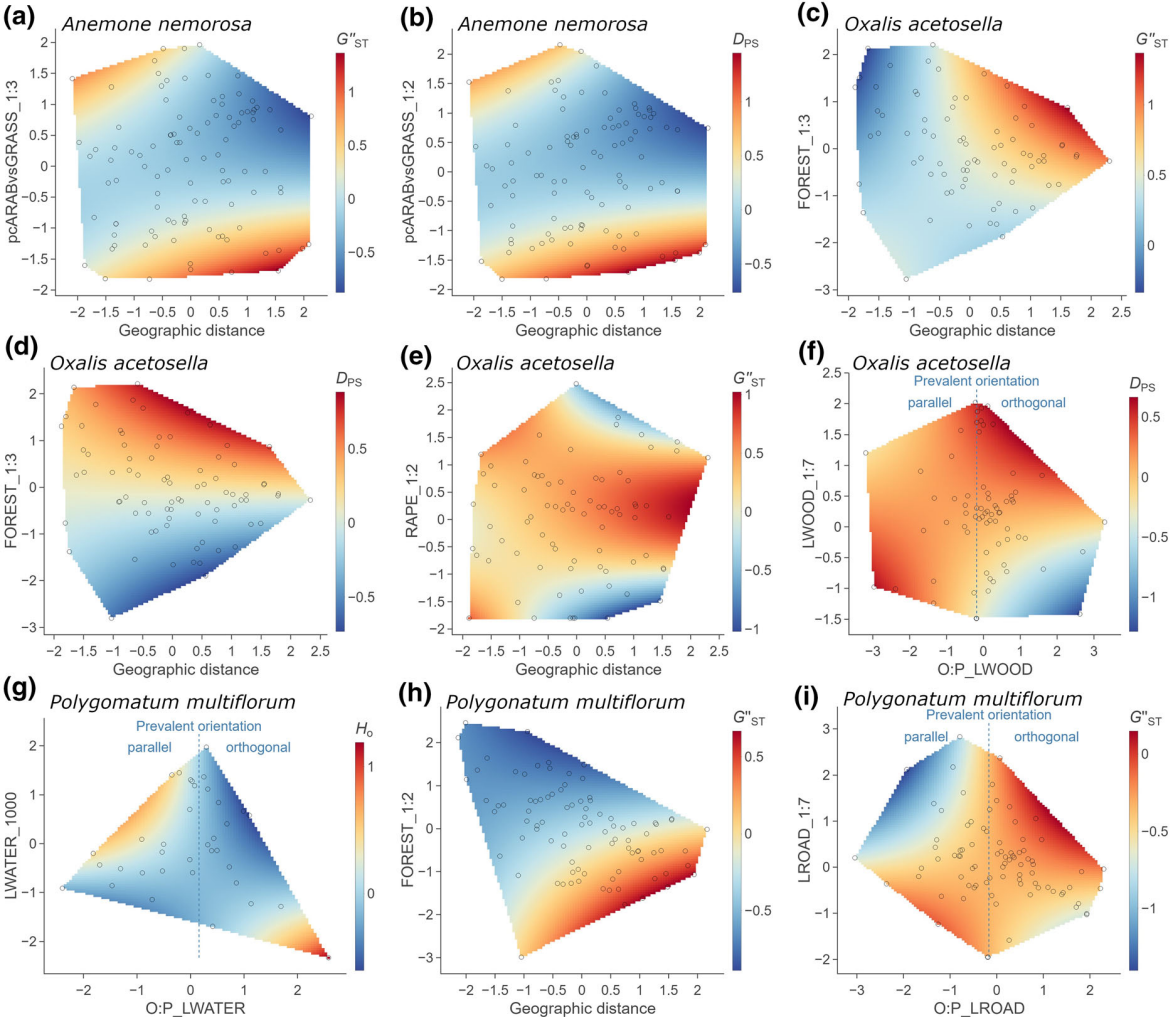
Visualizations of interactive effects (cf. Tables 2 and 3) of landscape metrics (x- and y-axis) on population genetic variables of *Anemone nemorosa*, *Oxalis acetosella* and *Polygonatum multiflorum* as heatmap. Only the area covered by the values of both explanatory variables (overlaid as points) is plotted. All variables are scaled in standard deviation units. Population genetic variables are measures of genetic differentiation (*D*_PS_ and *G’’*_ST_) and observed heterozygosity (*H*_O_). The landscape metrics FOREST and RAPE refer to the percent cover of deciduous forest and oilseed rape, respectively. LROAD, LWATER and LWOOD refer to the relative length of roads, water courses and hedgerows/treelines, respectively. The prefix O:P refers to orthogonal-to-parallel length ratio. pcARABvsGRASS is a principal component reflecting the trade-off between arable land (cereals and oilseed rape) on the one hand and grassland on the other hand (cf. Table 3). Numbers or ratios added to the variable names correspond to the most influential buffer distance in meters or the most influential width-to-length radio of the landscape strips, respectively

All important effects (importance value ≥ 0.5, see main text), are symbolized as follows: / positive effect, \ negative effect, X interactive effect (see Fig. 3), ∩ and ∪ unimodal effect with maximum and minimum, respectively, at an intermediate level of the landscape metric, /∩, \∩ and /∪ asymmetric unimodal effects with positive or negative trend, respectively. In addition, *R*^2^ values for each model are provided: marginal *R*^2^ (variation explained by fixed effects, i.e., jointly by basic population genetic determinants and landscape metrics), landscape *R*^2^ (variation explained uniquely by landscape metrics) and the percentage of the marginal R^2^ that can be uniquely attributed to landscape effects (%Landscape). See Table 1 for explanations on variable names. Numbers added to the variable names correspond to the most influential buffer distance in meters. See Table S6.1 for complete model results. Visualizations of the effects are presented in Figs. 2, 3, and S6.1 to S6.3

^a^Principal component from SETTLE_250 (*r* = 0.94) and LROAD_250 (*r* = 0.94)

^b^Principal component from SETTLE_500 (*r* = 0.93), LROAD_500 (*r* = 0.89) and EDGEDEN_500 (*r* = 0.90)

^c^Principal component from GRASS_1000 (*r* = 0.94) and LWOOD_1000 (*r* = 0.94)

^d^Principal component from CEREAL_2000 (*r* = 0.97), RAPE_2000 (*r* = 0.90), and GRASS_2000 (*r* = − 0.95)

^e^Principal component from SETTLE_1000 (*r* = 0.95) and LROAD_1000 (*r* = 0.95)

Some of the landscape metrics had contrasting effects for different species. With increasing cover of arable land, allelic richness of *A. nemorosa* increased, but decreased for *P. multiflorum* (Fig. 2a and k). Expected heterozygosity of *A. nemorosa* increased with increasing maize cover in the landscape, whereas for *O. acetosella*, expected heterozygosity was highest at a lower-than-average maize cover (Fig. 2d and h). Expected and observed heterozygosity of *A. nemorosa* also appeared to benefit from a high settlement cover in the landscape, while expected and observed heterozygosity of *P. multiflorum* decreased with increasing settlement cover (Fig. 2c, e, m, and n).

The differentiation of crop types resulted mostly in higher marginal *R*^2^-values than considering arable land in general, even though often only a single crop type yielded importance (Table 2a, b) or several crop types were highly correlated and were thus united in a principal component (Table 2c). All crop types had unique effects. The inbreeding coefficient of *A. nemorosa* populations was highest with an oilseed rape cover slightly above the mean (Fig. 2f). As stated above, maize cover affected the expected heterozygosity of *A. nemorosa* and *O. acetosella* differently. Allelic richness and expected heterozygosity of *P. multiflorum* populations decreased with increasing cover of cereals and oilseed rape, which was, however, at the same time negatively correlated with grassland cover (Table 2; Fig. 2k).

Linear landscape elements affected genetic diversity mostly independent of their prevalent orientation relative to the gene dispersal direction, with one exception (Fig. 3g): water courses orthogonal to the gene flow direction reduced observed heterozygosity in *P. multiflorum* populations, while water courses pointing towards the populations enhanced observed heterozygosity.

The spatial scale, at which the species’ genetic diversity responded to the landscape structure, was variable both across genetic diversity variables and landscape metrics (Table 2). Often, landscape effects were similarly high at several buffer distances (results not shown). For *P. multiflorum*, the majority (75%) of important landscape effects were most pronounced at a buffer distance ≥ 1000 m. No clear pattern occurred for the other two species.

### Landscape effects at the link level

Twelve landscape metrics were involved in at least one important landscape effect on a genetic differentiation measure (Table 3). The proportion of variation explained uniquely by the landscape effects generally exceeded 90% of the total variation explained by fixed effects. This means that geographic distance alone had little explanatory power. It determined, however, the magnitude and direction of several landscape effects (Fig. 3).

**Table 3:** Summary of landscape effects on measures of pairwise genetic differentiation (*G’’*_ST_ and *D*_PS_) as resulting from MLPE models at the link level, separately for (a) *Anemone nemorosa*, (b) *Oxalis acetosella*, and (c) *Polygonatum multiflorum*

Landscape metrics	*G''*_ST_	*D*_PS_
(a) *Anemone nemorosa*		
FOREST_1:3	/	∩
pcARABvsGRASS_1:3^a^	X	
pcARABvsGRASS_1:2^b^		X
LFRINGE_1:3	/	/
Marginal *R*^2^	0.16	0.25
Landscape *R*^2^	0.16	0.25
% Landscape	99.2	100.0
(b) *Oxalis acetosella*		
FOREST_1:3	X	X
ARABLE_1:3		/
RAPE_1:2	X	
LWOOD_1:7		X
LWATER_1:3	\	
LROAD_1:5	\	
SHANNON_2:3		\
Marginal *R*^2^	0.11	0.10
Landscape *R*^2^	0.11	0.09
% Landscape	99.9	99.4
(c) *Polygonatum multiflorum*		
FOREST_1:2	X	
SEMNATGRASS_1:3	/U	/
ORCHARD_2:3		/
LWOOD_1:5		\
LROAD_1:7	X	
LROAD_1:2		\
Marginal *R*^2^	0.18	0.33
Landscape *R*^2^	0.17	0.30
% Landscape	95.7	91.4

All important effects, i.e., those with an importance value ≥ 0.5 (see main text), are symbolized as follows: / positive effect, \ negative effect, X interactive effect (see Fig. 3), ∩ and ∪ unimodal effect with maximum and minimum, respectively, at an intermediate level of the landscape metric, /∩, \∩ and /∪ asymmetric unimodal effects with positive or negative trend, respectively. In addition, *R*^2^ values for each model are provided: marginal *R*^2^ (variation explained by fixed effects, i.e. jointly by basic population genetic determinants and landscape metrics), landscape *R*^2^ (variation explained uniquely by landscape metrics) and the percentage of the marginal *R*^2^ that can be uniquely attributed to landscape effects (%Landscape). See Table 1 for explanations on variable names. The ratio added to each variable name corresponds to the most influential width-to-length radio of the landscape strips. See Table S6.2 for complete model results. Visualizations of the effects are presented in Figs. 2, 3, and S6.1 to S6.3

^a^Principal component from CEREAL_1:3 (*r* = 0.95), RAPE_1:3 (*r* = 0.90) and GRASS_1:3 (*r* = − 0.92)

^b^Principal component from CEREAL_1:2 (*r* = 0.96), RAPE_1:2 (*r* = 0.90) and GRASS_1:2 (*r* = − 0.92)

Two landscape metrics had contrasting effects for the different species, forest cover and arable land. Genetic differentiation among *A. nemorosa* populations measured by *D*_PS_ was highest with an intermediate forest cover (Fig. 2g), while for *O. acetosella*, *D*_PS_ was highest with a high forest cover (Fig. 3d). When distance between populations was short, a high forest cover reduced *G’’*_ST_ among both *O. acetosella* and *P. multiflorum* populations. However, when distance between population was far, a high forest cover enhanced *G’’*_ST_ among *O. acetosella* populations, but reduced it among *P. multiflorum* populations (Fig. 3c and h). A high cover of arable land generally increased *D*_PS_ between *O. acetosella* populations (Fig. 2j), whereas for *A. nemorosa*, it either increased or decreased *D*_PS_ depending on whether the distance between populations was short or far, respectively (Fig. 3b).

Of the different crop types, only oilseed rape had a unique effect (Fig. 3e). For short distances between *O. acetosella* populations, *G’’*_ST_ was highest, when oilseed rape cover was high. With far distances between *O. acetosella* populations, *G’’*_ST_ was highest, when oilseed rape cover was intermediate, but lowest when oilseed rape cover was either very high or low.

Two of the eight effects of linear landscape elements depended on the orientation of the landscape elements relative to the landscape strip (Table 3). For *O. acetosella*, woody linear elements running parallel to the landscape strip had little effect, but those running orthogonal to the landscape strip enhanced *D*_PS_ among populations (Fig. 3f). For *P. multiflorum*, roads running parallel to the landscape strip reduced *G’’*_ST_ among populations, whereas those running orthogonal to the landscape strip enhanced *G’’*_ST_ (Fig. 3i).

The width-to-length ratio of the landscape strips, at which genetic differentiation among populations was influenced most, was variable (Table 3). There was no clear difference among species. Most landscape effects (81%) were most pronounced at an intermediate width-to-length ratio (1:5 to 1:2).

## Discussion

Our results show that gene flow among spatially isolated forest herb populations in agricultural landscapes is influenced by a multitude of different land-use types and landscape elements that act at different spatial scales. The composition and configuration of the landscape prove here to be more important determinants of the forest herbs’ landscape-scale population genetic structure than the size of local populations and their geographic distance to each other.

### Forest herbs respond differently to the landscape structure (H1)

Each species not only responded to a different set of landscape metrics, but also showed contrasting responses when affected by the same landscape metric (Tables 2 and 3). These contrasting responses were most pronounced for the cover of forest, arable land and settlements. Forest cover in landscape strips among populations affected the genetic differentiation among populations of all three species. For *O. acetosella*, a high forest cover in the landscape increased genetic differentiation among populations, particularly when distances among populations were far (Fig. 3c and d), indicating that forest hampers far-distance gene dispersal. While forests do not represent insurmountable barriers for bees, they appear to enhance landscape resistance for forage flights (Kreyer et al. 2004; Goulson et al. 2010; Kamm et al. 2010; Zurbuchen et al. 2010). In contrast, *P. multiflorum* showed the lowest genetic differentiation among populations, when forest cover was high (Fig. 3h). This stands in contrast to the observation that forest increases landscape resistance to bumblebee flights (Kreyer et al. 2004; Goulson et al. 2010). In fact, one of the most important pollinators of *P. multiflorum*, *Bombus pascuorum* (Naaf et al. 2021), was found to use floral resources in forests and open habitats at similar rates and at similar distances from their nests (Kreyer et al. 2004). Thus, for this bumblebee species, forests do not represent barriers but even allow *B. pascuorum* to practice its typical trap line behaviour and to visit several *P. multiflorum* populations on a single forage flight (Kreyer et al. 2004). Moreover, there is ample evidence that seed dispersal by both woodland birds (Heikkinen et al. 2004; Garcia et al. 2010) and carnivores (Herrera et al. 2016) is enhanced with a high forest cover. Finally, genetic differentiation among *A. nemorosa* populations was highest with an intermediate forest cover in the landscape (Table 3; Fig. 2g). This relationship might reflect a trade-off between effects that limit and promote gene flow. As mentioned above, a high forest cover might restrict bee movement through the landscape. However, it allows also a high richness and abundance of forest-dwelling hoverflies (Meyer et al. 2009; Schirmel et al. 2018; Proesmans et al. 2019a, b) and a short distance to other *A. nemorosa* populations.

Besides forest, the cover of arable land affected the species’ population genetic structure differently. While a high cover of arable land appeared to facilitate gene flow among *A. nemorosa* populations (Figs. 2a and 3b), it apparently restricted gene flow among populations of *O. acetosella* (Fig. 2j) and *P. multiflorum* (Fig. 2k). One gene-dispersal vector for *A. nemorosa* that benefits from arable land are aphidophagous hoverflies. Many hoverflies, which prefer forests and hedgerows as their adult habitat, prefer cropland as their larval habitat, where they feed on aphid colonies (Meyer et al. 2009). In fact, several studies found positive relationships between the abundance and species richness of aphidophagous hoverflies and the proportion of arable land in the landscape (Jauker et al. 2009; Meyer et al. 2009; Haenke et al. 2014). Thus, high abundances of aphidophagous hoverfly species, such as *Melanostoma scalare*, *Platycheirus albimanus* or *Syrphus ribesii*, which are important pollinators of *A. nemorosa* (personal observations), might be responsible for the positive effect of arable land on allelic richness and its negative effect on genetic differentiation among *A. nemorosa* populations. This is apparently not true for *O. acetosella*, for which genetic differentiation among populations increased with increasing cover of arable land in the landscape. Since the most dominant crop types, i.e., cereals and maize (Supp. Inf. S4), do not provide any floral resources during spring, they occur at the cost of more valuable habitats for pollinators, such as grasslands (Jakobsson and Ågren 2014; Bartual et al. 2019). The trade-off between arable land and grassland was even clearer for *P. multiflorum*, for which allelic richness and expected heterozygosity decreased with increasing cover of arable land at the cost of grassland (Table 2; Fig. 2k). Many bumblebee species benefit from a high grassland cover in the landscape because grasslands may provide both nesting sites and floral resources (Goulson et al. 2010; Vray et al. 2019). Also, the occupancy of forest patches by woodland birds appears to be positively affected by the amount of grassland in contrast to the amount of arable fields in the surrounding landscape (Radford and Bennett 2007; Montague-Drake et al. 2009).

Moreover, settlements and roads affected the forest herbs differently. Close to villages, the road network is particularly dense. Therefore, the effects of settlement area and road density were often confounded (Table 2). For *P. multiflorum*, the lower genetic diversity in populations less than 1 km away from settlements (Figs. 2l–n) implies that here, the inflow of new alleles via pollen or seeds occurs at lower rates than at far distance from settlements. Two mechanisms, corresponding to different gene dispersal vectors, might explain this pattern. First, woodland birds might avoid landscapes with more settlements and roads (Dunford and Freemark 2005; Rüdisser et al. 2015) and therefore transport *P. multiflorum* seeds less often into nearby forest patches. However, woodland birds were found to respond most strongly to landscape composition at a smaller spatial scale of 100 to 500 m (Rüdisser et al. 2015). Second, settlements in our agricultural landscapes consist mostly of rural villages with many gardens. Gardens are important forage habitats for bumblebees in rural landscapes (Goulson et al. 2010; Nakamura and Kudo 2019). The flowering period of fruit trees such as *Malus* spp., *Pyrus* spp. or *Prunus* spp. as well as ornamental shrubs such as *Syringa vulgaris* overlaps with that of *P. multiflorum*. These ample floral resources might attract bumblebees and reduce bumblebee abundance and their pollination service in nearby forest patches (Nakamura and Kudo 2019; Proesmans, et al. 2019a, b). Interestingly, at a smaller spatial scale (250 m), the inbreeding coefficient for *P. multiflorum* was negatively related to settlement area (Fig. 2o), indicating that *P. multiflorum* populations very close to villages might benefit from bumblebee spill-over (Cussans et al. 2010). In contrast to *P. multiflorum*, *A. nemorosa* appeared to benefit from settlement areas in the landscape (250–500 m), at least in terms of expected and observed heterozygosity (Figs. 2c and e). Close to villages, different foraging, resting or nesting habitats for pollinators, such as gardens and hedgerows, have a relatively high density. They might sustain a high abundance of pollinators (Cussans et al. 2010; Garratt et al. 2017; Schirmel et al. 2018; Bartual et al. 2019), which then spill over to nearby forest patches to feed on pollen of *A. nemorosa* in early spring.

In general, there was no clear difference in the spatial scale, at which the three species responded to the landscape structure. The prevalent buffer distances ≥ 1000 m for *P. multiflorum* reflect its association with far-flying bumblebees. Important pollinator species such as *Bombus pascuorum* and *B. pratorum* (Naaf et al. 2021) may forage over distances > 1800 m (Redhead et al. 2016) and > 670 m (Knight et al. 2005), respectively.

### Distinguishing crop types matters (H2)

In nine out of 11 landscape effects, in which arable crops were involved (Tables 2 and 3), a differentiation of crop types resulted in higher proportions of explained variation than merging the different crop types into arable land in general. In particular oilseed rape and maize had distinct effects, which is remarkable considering that both crop types represent relatively young elements in European landscapes that were more or less absent seven decades ago (Knoema 2020). There was thus limited time for them to leave their imprint in the forest herbs’ population genetic structure. We had expected to find any effects of oilseed rape or maize most likely for *O. acetosella* given its shorter generation time compared to *A. nemorosa* and *P. multiflorum* (Naaf et al. 2021). Indeed, oilseed rape affected the genetic differentiation among populations of *O. acetosella* (Fig. 3e), while maize influenced genetic diversity within its populations (Fig. 2h and i). The high genetic differentiation among *O. acetosella* populations separated by a matrix of high oilseed rape cover might reflect a dilution effect (Holzschuh et al. 2011, 2016). The flowering periods of oilseed rape and *O. acetosella* greatly overlap and oilseed rape is highly attractive for various pollinators (Haenke et al. 2014; Riedinger et al. 2015). The attraction of pollinators by oilseed rape might lead to diluted pollinators in adjacent forests (Holzschuh et al. 2011; Van Reeth et al. 2019), where *O. acetosella* plants might receive little compatible pollen from other forest patches. However, when oilseed rape cover in the landscape strip was very high and distances between populations were > 2300 m, we observed a reduced genetic differentiation among *O. acetosella* populations (Fig. 3e). In this case, the landscape strip crossed large parts of the landscape window. A permanently high oilseed rape cover across the landscape window might result in positive population growth rates of pollinators and thus higher abundances in the long term. For instance, the abundance of solitary bees was found to be enhanced by a high oilseed rape cover in the preceding year (Riedinger et al. 2015).

The negative effect of maize cover on expected heterozygosity of *O. acetosella* populations (Fig. 2h) indicates that a high cover of maize reduces the inflow of new alleles. Also the inbreeding coefficient of *O. acetosella* populations increased with maize cover, potentially reflecting pollen limitation (Fig. 2i). However, this increase levelled off and changed into a decrease when maize cover was very high. Since pollen limitation is unlikely to decrease, when maize cover is very high, enhanced seed dispersal might prevent inbreeding under these circumstances. Maize fields are a very attractive forage and shelter habitat for wild boar during summer and autumn (Keuling et al. 2009; Tillmann 2011), when the cleistogamous seeds of *O. acetosella* are ripe (Berg and Redbo-Torstensson 1998). Some of these animals appear to move regularly between the maize fields and forests (Keuling et al. 2009) and might thus disperse seeds of *O. acetosella*.

Despite its longer generation time, also *A. nemorosa* showed some responses to oilseed rape and maize. Populations with a high oilseed rape cover in the surrounding landscape showed an increased inbreeding signal (Fig. 2f). Although *A. nemorosa* starts flowering earlier than oilseed rape, there might be an overlapping flowering period of one or two weeks. Thus, as with *O. acetosella*, the increased inbreeding signal might result from pollinator dilution (Holzschuh et al. 2011; Van Reeth et al. 2019). The positive effect of maize cover on expected heterozygosity of *A. nemorosa* populations (Fig. 2d) was unexpected (Table 1) and difficult to explain. Given the young history of maize in our landscapes in relation to the long generation time of *A. nemorosa*, this result should be considered with caution.

### The orientation of linear landscape elements matters (H3)

Our results show that linear landscape elements, including woody line elements, water courses, herbaceous fringes and roads, influence the movement of gene dispersal vectors across the landscape. The orientation of the linear landscape elements was important in three out of 16 effects (Tables 2 and 3). Here, the linear landscape elements appeared to have a promoting or hampering effect on gene flow as hypothesized, depending on whether they were predominantly oriented parallel or orthogonal to the gene dispersal pathways (Fig. 3f, g, and i). These results confirm the observation that linear landscape elements may not just channel animal movements, but may also act as relative barrier (Wratten et al. 2003; Levey et al. 2005; Saïd and Servanty 2005; Krewenka et al. 2011; Klaus et al. 2015; Fitch and Vaidya 2021).

At large spatial scales (2000 m radius around populations), orthogonal and parallel orientations of linear landscape elements can be expected to be balanced. For this situation, we found a strong positive effect of road density on the inbreeding coefficient of *P. multiflorum* populations, indicating that roads restrict outbreeding (Table 2; Fig. 2p). Whether this effect is mediated through pollen or seed dispersers or both remains unclear. Bumblebees as well as many rodents seem to be reluctant to cross roads (Trombulak and Frissell 2000; Bhattacharya 2003).

Also at the 2000 m scale, there was a strong unimodal response of allelic richness in *A. nemorosa* populations to the density of woody linear elements (Table 2; Fig. 2b). These appeared to promote allelic richness up to an above-average density in the landscape. This effect might result from a higher abundance of pollinators in landscapes with a high hedgerow density and spill-over from hedgerows to adjacent forests. Hedgerows represent important resting and foraging habitats for both hoverflies (Haenke et al. 2014; Garratt et al. 2017; Schirmel et al. 2018) and bees (Garratt et al. 2017; Bartual et al. 2019). The subsequent decrease in allelic richness at an even higher density of woody linear elements was mainly due to the French populations (Fig. 2b). On the one hand, this decrease could result from an increasing resistance that woody linear elements constitute for pollinator movements (Wratten et al. 2003; Krewenka et al. 2011; Klaus et al. 2015). This interpretation would be analogous to that of the unimodal effect of forest cover on genetic differentiation among *A. nemorosa* populations (see above, Fig. 2g). On the other hand, the high hedgerow density in the French landscape window is rather recent and established only in the nineteenth century (Jamoneau et al. 2012). Thus, the time passed since then might not suffice for the hedgerows to leave their imprint in the genetic diversity of *A. nemorosa* populations.

## Conclusions

Our study shows that, more than habitat loss and fragmentation per se, the composition and configuration of the agricultural landscape matrix exerts significant control over the population genetic structure, and thus the functional connectivity, in fragmented temperate forest herb populations. Although we could only discuss rather than reveal the underlying mechanisms of the observed landscape effects, it became obvious that a multitude of mechanisms are at work in our landscapes. In this respect, our study has generated many hypotheses, which deserve to be tested in depth. The observed landscape effects turned out to be rather species-specific. Nevertheless, they are general in the sense that they were consistent across multiple agricultural landscapes across Europe.

Landscape planning with the aim to enhance the functional connectivity among spatially isolated plant populations in agricultural landscapes should consider that (a) species of the same ecological guild (e.g., forest herbs) might respond quite differently to the landscape structure, if they are associated with different pollen or seed dispersal vectors; (b) it may be worth to differentiate crop types rather than merging them into arable crops in general given their distinct effects on functional connectivity; and (c) linear landscape elements that are mostly perceived as relative barriers for animals, such as roads, may also have a channelling effect on their movement and thus promote gene flow, whereas linear landscape elements that are mostly perceived as connecting corridors, such as hedgerows, may also act as barrier for gene dispersal vectors.

## Supplementary Information

Below is the link to the electronic supplementary material.

Supplementary file1 (PDF 4969 kb)

## Data Availability

The microsatellite allele tables for all species and populations as well as population locations are available on DRYAD (https://doi.org/10.5061/dryad.tb2rbp00k). The population genetic variables at the node level are published in Naaf et al. 2021 (https://doi.org/10.1007/s10980-021-01292-w). The population genetic variables at the link level and all landscape metrics (node and link level) are available on DRYAD as well (https://doi.org/10.5061/dryad.h70rxwdkf).
