# Supplementary material for: Context matters: the landscape matrix determines the population genetic structure of temperate forest herbs across Europe
Source: Landsc Ecol. 2021 Dec 1;37(5):1365–84. doi: 10.1007/s10980-021-01376-7 (PMC9085688; doi:10.1007/s10980-021-01376-7)
Supplement: Supplementary file 1 — Supplementary file1 (PDF 4969 kb) [file 10980_2021_1376_MOESM1_ESM.pdf]

Landscape Ecology

Supplementary Information for

Context matters: the landscape matrix determines the population genetic structure of temperate forest herbs across Europe

Tobias Naaf*, Jannis Till Feigs, Siyu Huang, Jörg Brunet, Sara A. O. Cousins, Guillaume Decocq, Pieter De Frenne, Martin Diekmann, Sanne Govaert, Per-Ola Hedwall, Jonathan Lenoir, Jaan Liira, Camille Meeussen, Jan Plue, Pieter Vangansbeke, Thomas Vanneste, Kris Verheyen, Stephanie I. J. Holzhauer, Katja Kramp

* Corresponding author: Leibniz Centre for Agricultural Landscape Research (ZALF), Eberswalder Strasse 84, D-15374 Müncheberg, Germany; naaf@zalf.de

S1 Land-use maps of the seven landscape windows 2
S2 Descriptive statistics of population attributes 11
S3 Microsatellite markers used for genotyping and PCR protocols 12
S4 Crop dominance 15
S5 Additional information on the expected landscape effects shown in Table 1 in the main text ... 18
S6 Complete modelling results 25

S1 Land-use maps of the seven landscape windows

As a basis for our landscape analysis, we created digital land-use maps for all landscape windows including a 2 km buffer based on the most up-to-date aerial photographs as well as cloud-free Sentinel-2 satellite images and relevant national or regional additional data:

North France

- Orthophotos from 2013; source: Institut national de l'information géographique et forestière
- SPOT 6 satellite images from 2017; source: Institut national de l'information géographique et forestière
- Sentinel-2 scenes from 2017/05/26, 2017/07/05 and 2017/10/18

Belgium

- Orthophotos from 2015; source: Nationaal Geografisch Instituut (https://www.ngi.be/website/)
- Vegetation and land-cover map; source: Nationaal Geografisch Instituut (https://www.ngi.be/website/)
- Sentinel-2 scenes from 2017/05/06, 2017/05/26, 2017/10/15 and 2017/10/18

West Germany

- Color-infrared-orthophotos from 2018; source: Bundesamt für Kartographie und Geodäsie
- Environmental maps for Lower Saxony (conservation areas, habitat type maps, etc.); source: Niedersächsisches Ministerium für Umwelt, Energie, Bauen und Klimaschutz (https://www.umwelt.niedersachsen.de/startseite/service/umweltkarten)

East Germany

- Color-infrared-orthophotos from 2009 and 2016; source: Landesvermessung und Geobasisinformation Brandenburg
- Sentinel-2 scenes from 2017/05/27, 2017/07/09, 2017/08/15 and 2017/10/19
- Brandenburger Biotoptypen- und Landnutzungskartierung 2009 (Habitat type and land-use map); source: Landesamt für Umwelt Brandenburg
- Geschützte Biotope 2016 (Preserved habitats) 2016; source: Landesamt für Umwelt Brandenburg
- Referenzierte Moorkarte für das Land Brandenburg 2013 (Referenced Peatland Map for Brandenburg); source: Landesamt für Bergbau, Geologie und Rohstoffe Brandenburg
- Karte potenziell schutzwürdiger Moorböden in Brandenburg 2017 (Map of peatland soil of potential conservation value); source: Landesamt für Umwelt Brandenburg
- Conservation areas; source: Landesamt für Umwelt Brandenburg
- IACS data 2010-2017; source: Landesamt für Umwelt Brandenburg

South Sweden

- Color-infrared-orthophotos from 2016; source: Lantmäteriet
- Sentinel-2 scenes from 2017/05/27, 2017/06/09, 2017/08/08, 2017/08/15, 2017/09/24 and 2017/10/09

Central Sweden

- Color-infrared-orthophotos from 2017; source: Lantmäteriet
- Sentinel-2 scenes from 2017/05/04, 2017/05/27, 2017/07/06 and 2017/10/19

Estonia

- Orthophotos from 2016; source: Maa amet (https://geoportaal.maaamet.ee/)
- Sentinel-2 scenes from 2017/05/02, 2017/06/14, 2017/07/31, 2017/08/30 and 2017/09/29
- Environmental maps with information on peatland sites 2016; source: Maa amet (https://geoportaal.maaamet.ee/)
- Digital surface model 2016; source: Maa amet (https://geoportaal.maaamet.ee/)

Besides the land-use types commonly used in other studies, such as forest, grassland and arable land, we also mapped some land-use types that we considered important as foraging habitat for pollinators, i.e. traditional grassland orchards, semi-natural grassland and unsealed green settlement areas (incl. gardens) (Table S1). As linear landscape elements, we mapped hedgerows, tree lines, water courses, roads as well as broad herbaceous fringes (width > 3 m), which might serve as foraging or nesting habitat for pollinators. The following mapping standards were used:

- Minimum size for polygons: 0.2-0.5 ha; minimum width: 15 m
- Minimum length for lines: 50 m; maximum width: 15 m

The mapping of green areas was based on structural and color differences, as well as the context in comparison with the surrounding areas. Arable land is characterized by a very homogeneous and evenly cultivated growth. In particular, a specific width of lanes and the often large surface area indicate this type of use. The growth can appear comparatively high. Flower-rich areas show higher heterogeneity in color and texture, which indicates a less intensive management. Sometimes small patch size, as well as slight woody growth can indicate an extensive management. In contrast, intensively managed grassland is often characterized by narrow lanes, a homogeneous structure and/or animal tracks. These areas are usually easily accessible and located within intensively used agricultural areas. They are often very large in comparison with extensive grasslands. Grasslands were indicated as "not definable" if they neither appeared to be intensively managed, nor showed a high structural diversity or floweriness. Wet meadows are often located near rivers or lakes and have a darker color, as well as a high structural diversity. Also numerous ditches can indicate high moisture content.

Table S1 Distinguished landscape elements

Area-based land-use types	Linear landscape elements
• Forest<br>○ Deciduous<br>○ Coniferous<br>• Arable land<br>• Grassland<br>○ semi-natural (rich in flowers)<br>○ Intensively managed (poor in flowers)<br>○ not definable<br>• Other semi-natural vegetation (heathland, fens, ruderal vegetation)<br>• Traditional grassland orchards<br>• Settlement area<br>○ Sealed or built-up area<br>○ Unsealed, green area (gardens, parks)<br>• Water bodies<br>• Others	• Hedgerows and tree lines<br>• Herbaceous fringes (> 3 m width)<br>• Roads<br>• Water courses and draining ditches

The following land-use maps each comprise the 5 × 5 km² landscape window plus a 2 km buffer.

North France

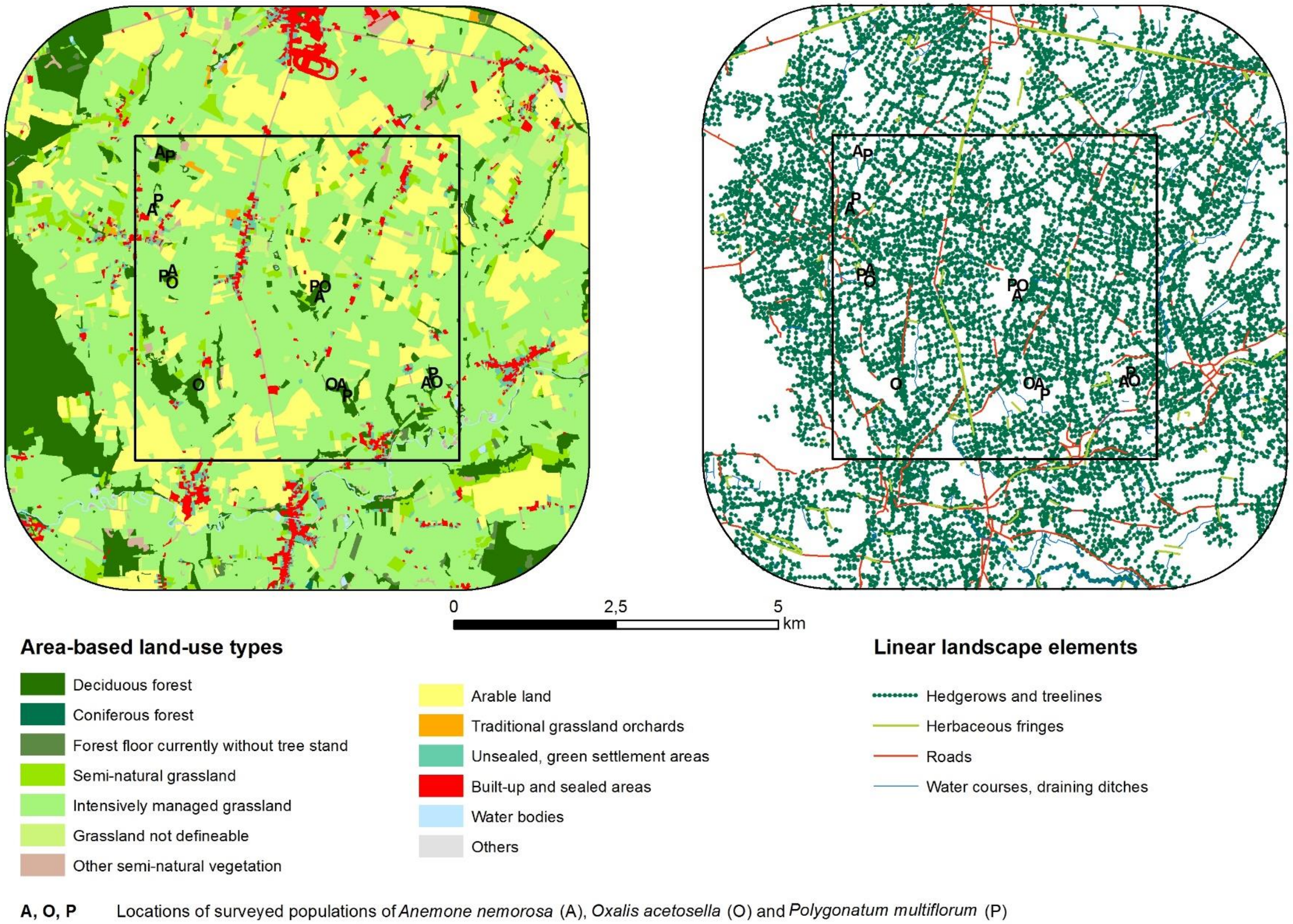

Belgium

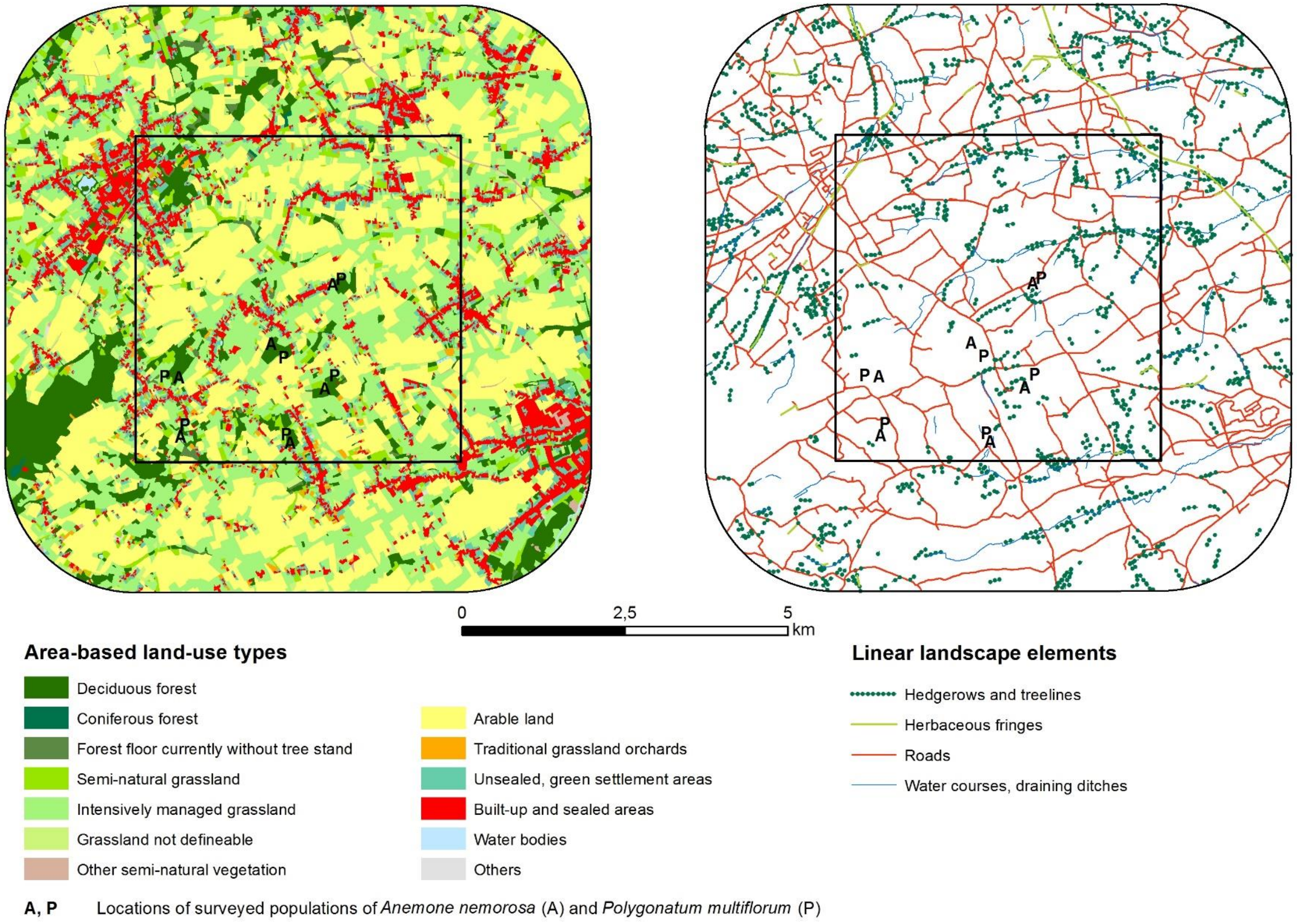
A
P
0
2,5
5
km
Area-based land-use types
Deciduous forest
Coniferous forest
Forest floor currently without tree stand
Semi-natural grassland
Intensively managed grassland
Grassland not defineable
Other semi-natural vegetation
Arable land
Traditional grassland orchards
Unsealed, green settlement areas
Built-up and sealed areas
Water bodies
Others
Linear landscape elements
Hedgerows and treelines
Herbaceous fringes
Roads
Water courses, draining ditches
A, P Locations of surveyed populations of *Anemone nemorosa* (A) and *Polygonatum multiflorum* (P)

West Germany

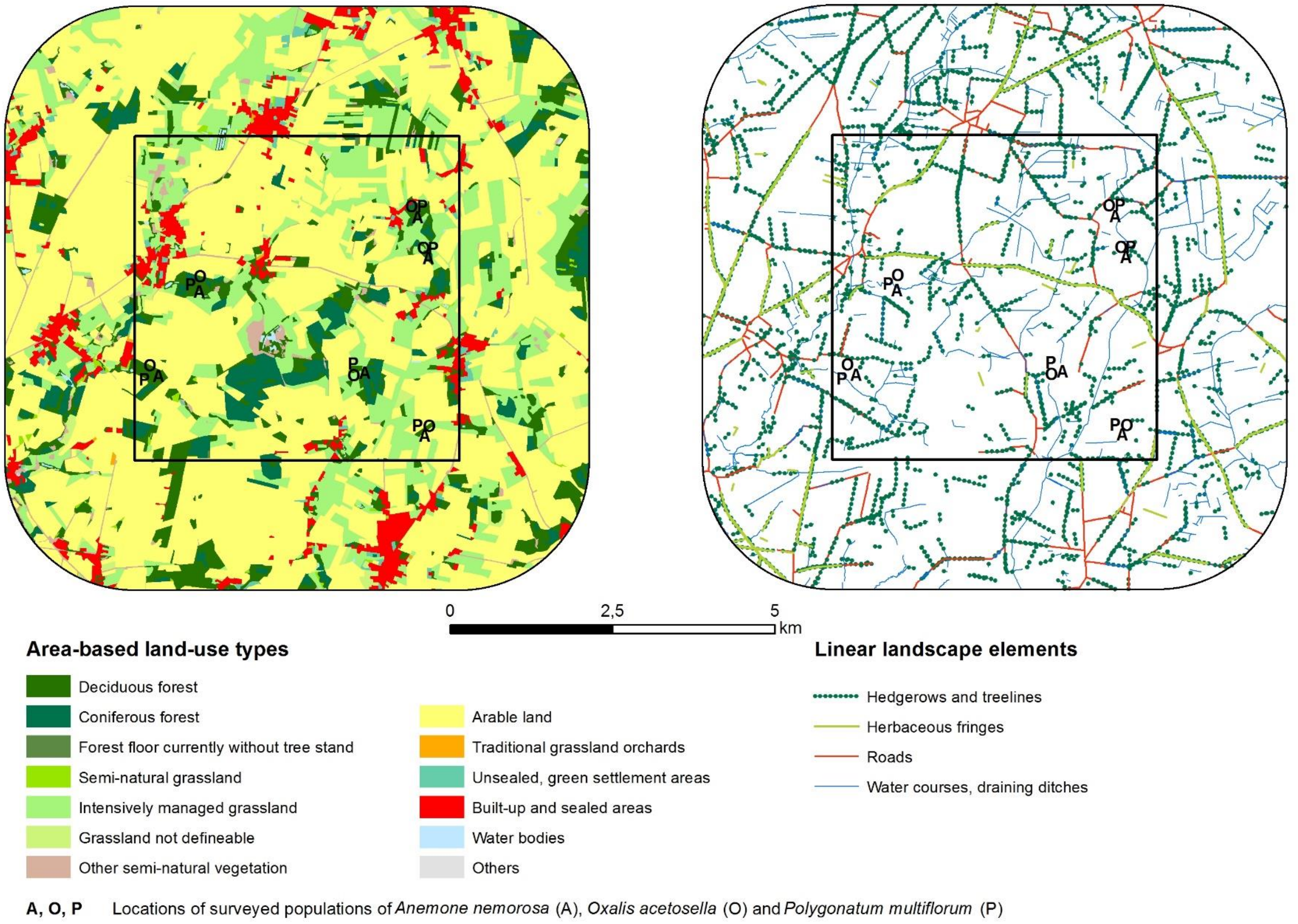

East Germany

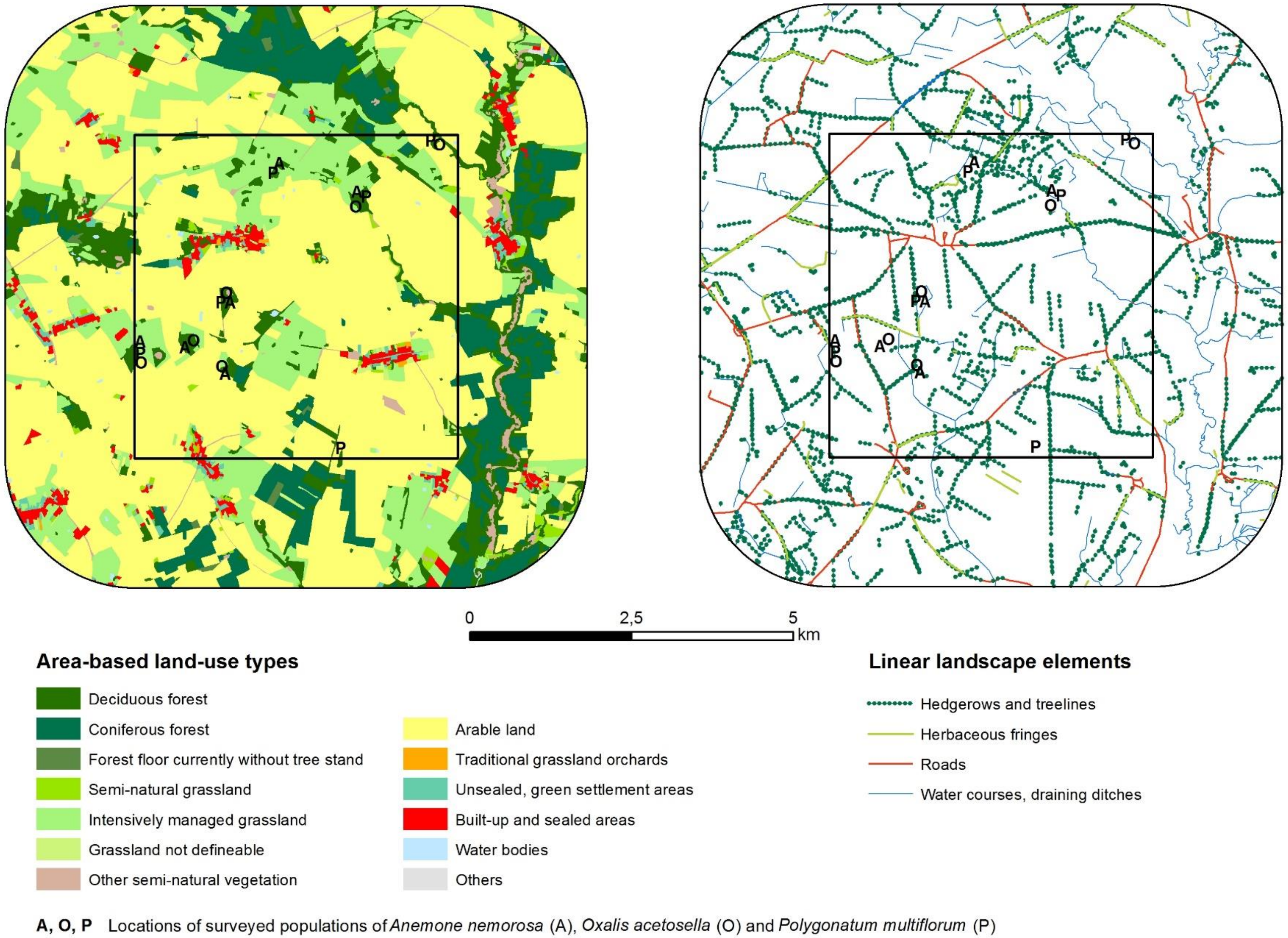

South Sweden

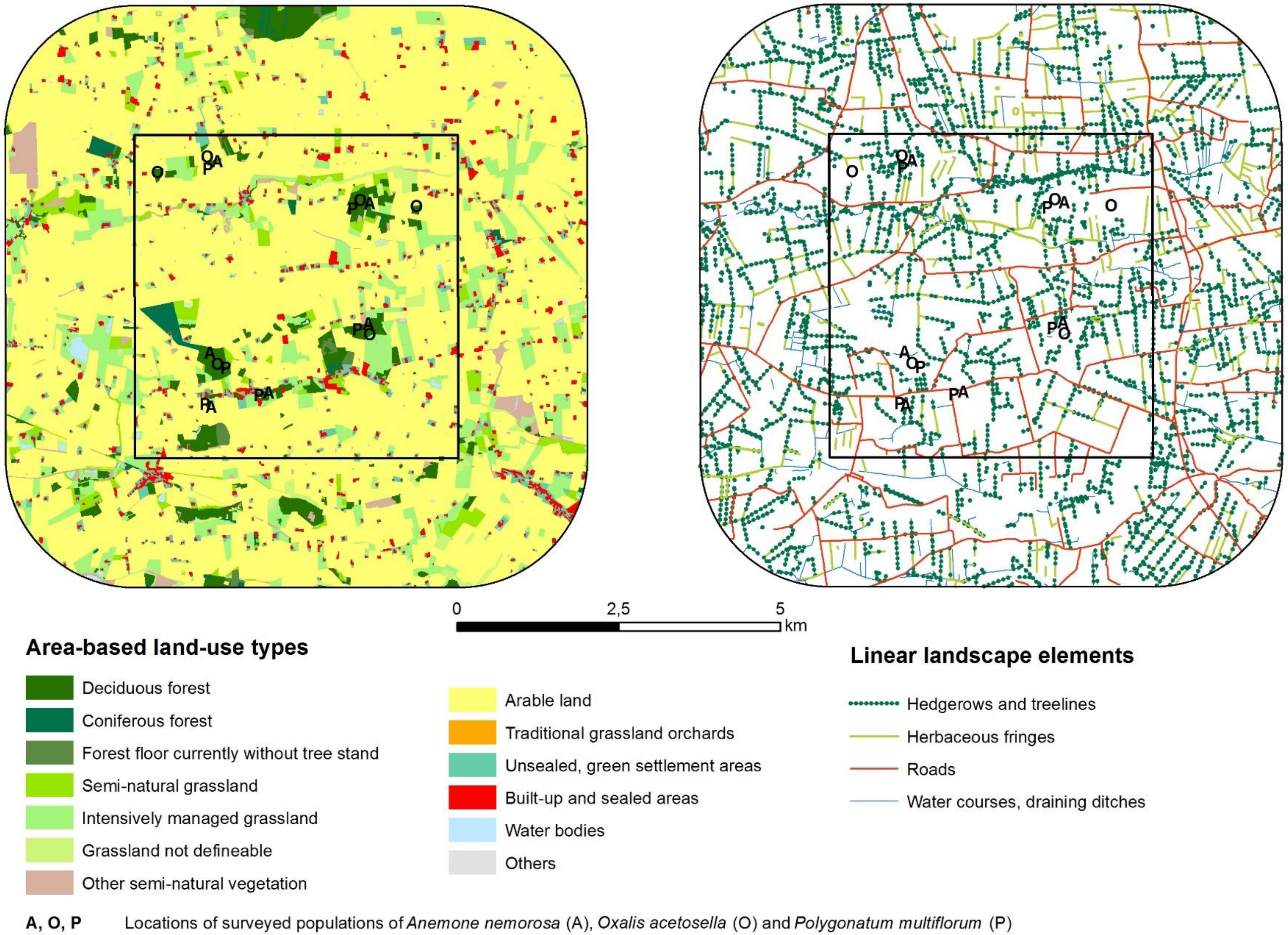

Central Sweden

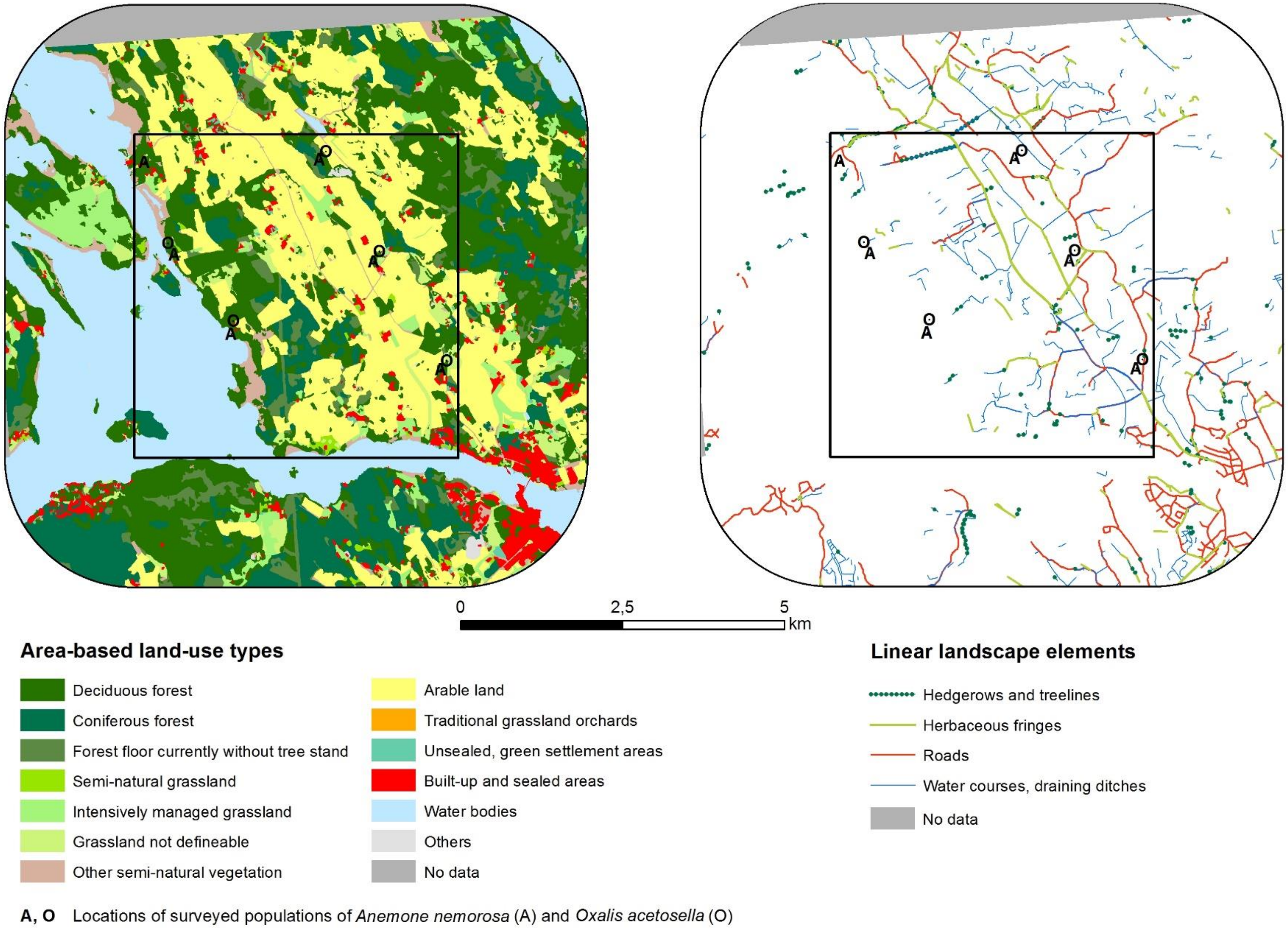

Estonia

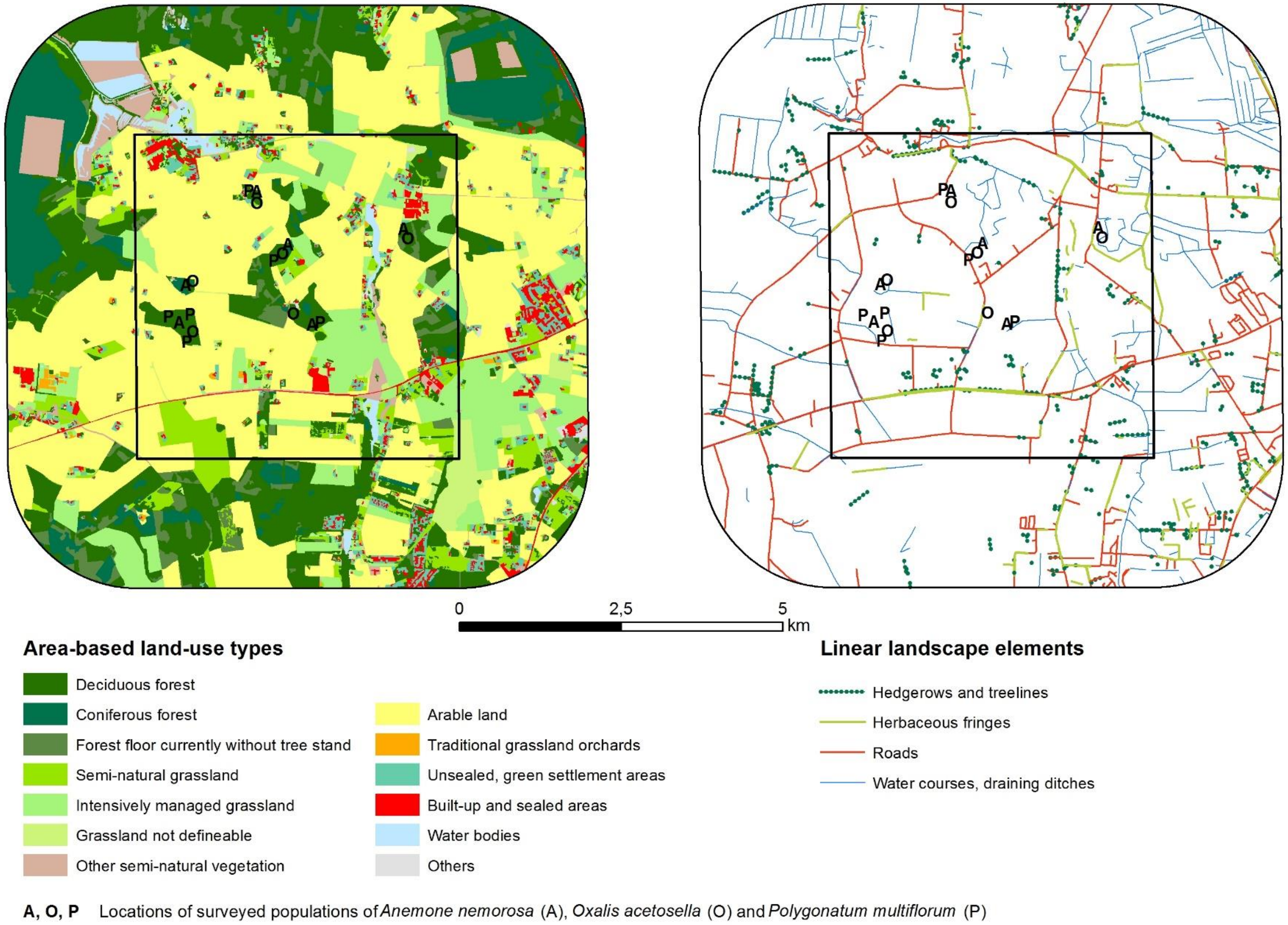
0
2,5
5
km
Area-based land-use types
Deciduous forest
Coniferous forest
Forest floor currently without tree stand
Semi-natural grassland
Intensively managed grassland
Grassland not defineable
Other semi-natural vegetation
Arable land
Traditional grassland orchards
Unsealed, green settlement areas
Built-up and sealed areas
Water bodies
Others
Linear landscape elements
Hedgerows and treelines
Herbaceous fringes
Roads
Water courses, draining ditches
A, O, P Locations of surveyed populations of Anemone nemorosa (A), Oxalis acetosella (O) and Polygonatum multiflorum (P)

S2 Descriptive statistics of population attributes

Table S2 Minimum (Min.), median and maximum (Max.) of basic population genetic determinants (population size, connectivity and geographic distance among pairs of populations), within-population genetic diversity (allelic richness (A_r), expected (H_e) and observed heterozygosity (H_o) and inbreeding coefficient (F)) and among-population genetic differentiation (G''_{ST} and D_{PS})

	Anemone nemorosa			*Oxalis acetosella*			*Polygonatum multiflorum*		
	Min.	Median	Max.	Min.	Median	Max.	Min.	Median	Max.
Population size	865	426853	12758494	70	75011	12820444	15	451	63204
Connectivity	12.2	605	7314	7.5	437	10360	0.6	30.1	79.7
Geogr. dist. (m)	306	1995	5263	424	2225	5518	214	2234	5518
A_r	3.8	7.5	9.7	1.1	2.5	3.2	4.3	7.9	10.5
H_e	0.56	0.72	0.77	0.04	0.39	0.54	0.56	0.74	0.79
H_o	0.37	0.51	0.66	0.04	0.30	0.56	0.56	0.78	0.88
F	0.13	0.29	0.40	-0.14	0.20	0.60	-0.25	-0.07	0.16
G''_{ST}	0.00	0.11	0.51	-0.03	0.17	0.84	0.07	0.25	0.50
D_{PS}	0.16	0.27	0.52	0.08	0.22	0.59	0.34	0.47	0.64

S3 Microsatellite markers used for genotyping and PCR protocols

Anemone nemorosa

Table S3.1 Microsatellite markers used to genotype samples of *A. nemorosa*. The markers were developed for *A. amurensis* by Sun et al. (2012)

Locus	Primer sequences (5'-3')	Repeat	Size (bp)	T_a (°C)	N_a
BH84	F: TTGCCATGGACCAATACTCG<br>R: GTCAGTGCAAGAAAGTAGCTGC	$(TG)_9$	161-203	48	22
BH206	F: TGTTGTTTCCCTTACTTGCC<br>R: CATCTTATGTCACACTTGGG	$(GT)_{22}A(TG)_{14}$	113-167	48	27
BH235	F: CATGGCCATTGGTATCAAAC<br>R: TTGGTGGAACAACTTAGCCC	$(GT)_5A(TG)_{16}$	144-190	48	22
HS27	F: GGAAGCATCATCTCACCTAC<br>R: TTCTAGTTTTGACTGGGAGG	$(AC)_7$	173-183	50	4
HS177	F: GAAAATGTGACCGTCCCTAC<br>R: TGTCATTGGCTCACCACCTT	$(AC)_7$	192-210	48	8
HS256	F: CTGTTCCTCCGATGGCGTTT<br>R: ACCTTACCCTTCCCCTCTTC	$(TG)_7$	214-254	50	19
				Mean	17.0
				Sum	102

Multiplex PCRs were performed in a final reaction volume of 15 µl, containing 0.5 µl of DNA (ca. 10-30 ng/µl), 7.5 µl of QIAGEN Multiplex PCR Plus Kit (100), 5.5 µl of H_2O and 1.5 of µl primer mix. Singleplex PCRs were performed in a final reaction volume of 10 µl, containing 0.5 µl of DNA (ca. 10-30 ng/µl), 5 µl of QIAGEN Multiplex PCR Plus Kit (100), 3.5 µl of H_2O and 1 µl primer mix. The primer mix for both singleplex and multiplex PCR contained 1 µl of each forward primer (labelled with fluorescent dye; stock solution concentration 100 pmol), 1 µl of each reverse primer and 98 µl of H_2O per 100 µl. A double PCR with the same primer set was done for samples with a low quality of DNA (A260/A230 < 1.5 and A260/A280 < 1.75) that did not result in countable banding patterns after a single PCR. Here, we conducted the first PCR as described above and a second PCR with the same conditions but with 0.5 µl of the PCR product of the first run as template.

For all loci, we applied the following standard PCR program:

Step	Initial denaturation	Denaturation	Annealing	Primer extension	final extension
Time (min)	5:00	0:30	**1:30**	**0:30**	10:00
T (°C)	95	95	T_a	72	68
		35 cycles			

Oxalis acetosella

Table S3.2 Microsatellite markers used to genotype samples of *O. acetosella*. The markers were developed by AllGenetics & Biology SL (www.allgenetics.eu) based on eight of our samples

Locus	Primer sequences (5'-3')	Repeat	Size (bp)	T_a (°C)	N_a
Oac111	F: CGTCATCTACACTCGTCGGA<br>R: GGCTAGGAGAGGTCGGAGTC	$(AG)_7$	172-178	57/53*	7
Oac113	F: TCCATCATCTCACACGCTTC<br>R: TTTGCTGGTGAAATGACGAC	$(AG)_6$	218-224	57/53	3
Oac114	F: TGGCACCATGTCATCATCTT<br>R: TTGTATTGTCGTGGACGGAG	$(AG)_8$	112-118	57/53	3
Oac159	F: CCCTGGTATCACGCATTTCT<br>R: AGGTGGTGTCTGTGGAGGAT	$(AG)_{10}$	124-164	57/53	13
Oac167	F: CCAAGAAATTCGGGTTGTTG<br>R: CTTACACGTTGCTCCTCCGT	$(AAG)_6$	166-181	57/53	6
Oac181	F: CCTTAGCAAGCTCCATCACC<br>R: GTTCTGTGCTTAATGCGACG	$(AG)_8$	130-134	57/53	3
Oac306	F: GTCAGTGCCACATCAGCTTG<br>R: CCGTAAGAAACGGATCCAAC	$(AC)_8$	201-225	57/53	2
Oac450	F: TCGCTAATGCGCAGATTTC<br>R: CATGCGCCTTTGCATTATTA	$(AAG)_9$	150-265	57/53	22
Oac466	F: CGATCAATCTGCGACAAGAA<br>R: GGAGAGTCGGTGGGAGTTC	$(AG)_6$	112-123	57/53	2
				Mean	6.8
				Sum	61

*See PCR protocol below for applied annealing temperatures.

For all loci, PCRs were performed in a final reaction volume of 12.5 µl, containing 1 µl of DNA (ca. 50-100 ng/µl), 6.25 µl QIAGEN Multiplex PCR Plus Kit (100), 4 µl of H_2O and 1.25 µl of primer mix. The primer mix for a singleplex PCR contained 2 µl of forward primer (stock solution concentration 100 pmol), 0.2 µl of reverse primer (with an oligonucleotide tail at its 5' end), 2 µl of fluorescent-labelled oligonucleotide (identical to the 5' tail of the reverse primer) and 95.8 µl of H_2O per 100 µl.

The oligonucleotide tails used were the universal sequences M13 (GGA AAC AGC TAT GAC CAT), CAG (CAG TCG GGC GTC ATC), and T3 (AAT TAA CCC TCA CTA AAG GG). The three oligonucleotides were labelled with the HEX dye, the FAM dye, and the TAMRA dye, respectively. During the first cycles of the PCR, the reverse primer with the tail is incorporated into the accumulating PCR products. When this primer is used up, the annealing temperature is lowered, so the fluorescently labelled M13, CAG, or T3 oligonucleotide can anneal and start acting as a primer.

For all loci, we applied the following standard PCR program:

Step	Initial denaturation	Denaturation	Annealing	Primer extension	Denaturation	Annealing	Primer extension	final extension
Time (min)	5:00	0:30	1:30	0:30	0:30	1:30	0:30	15:00
T (°C)	95	95	57	72	95	53	72	68
		35 cycles			8 cycles			

Polygonatum multiflorum

Table S3.3 Microsatellite markers used to genotype samples of *P. multiflorum*. The markers were developed for *P. cyrtonema* by Cheng et al. (2010) and for *P. filipes* by Liu et al. (2010)

Locus	Primer sequences (5'-3')	Repeat	Size (bp)	T_a (°C)	N_a	Source
Pc1	F: CTCTCCTATCGGCAGCAACT<br>R: ACTTCCTCCATCCTTACACCAT	$G_8(GA)_{32}$	184-198	54	6	Cheng et al. 2010
Pc17	F: GGACACCCGAAGAAATACAAG<br>R: CCAATTGCCTCCTTCACATC	$(AG)_{40}$	146-242	52	44	Cheng et al. 2010
Pc25	F: CTCCCTTTCCCAATCCCGT<br>R: CCCAACATCTCGTAGTCGCAA	$(CT)_8CC(CT)_{18}(TA)_5$	210-262	52	24	Cheng et al. 2010
Pc33	F: CGCACCCAGACCGAGAAA<br>R: GTAGGCAAGGAACACCCACAC	$(GA)_{34}$	228-280	54	25	Cheng et al. 2010
Pt9	F: ATGATGAGACCATAGGCGACT<br>R: GACGACTACGATGTCACCG	$(GA)_{39}$	126-216	54	45	Liu et al. 2010
Pt11	F: GGGGCTGCTGCTAGGGTAT<br>R: TCGCCTGTCACTGGATTGC	$(GA)_{26}$	135-187	54	5	Liu et al. 2010
				Mean	24.8	
				Sum	149	

For all loci, PCRs were performed in a final reaction volume of 15 µl, containing 1 µl of DNA (ca. 10-30 ng/µl), 7.5 µl of QIAGEN Multiplex PCR Plus Kit (100), 5 µl of H_2O and 1.5 of µl primer mix. The primer mix for a singleplex PCR contained 1µl of forward primer (labelled with fluorescent dye; stock solution concentration 100 pmol), 1 µl of reverse primer and 98 µl of H_2O per 100 µl.

For all loci except Pc17 and Pt9, we applied the following standard PCR program:

Step	Initial denaturation	Denaturation	Annealing	Primer extension	final extension
Time (min)	5:00	0:30	**1:30**	**0:30**	15:00
T (°C)	95	95	T_a	72	68
		35 cycles			

For Pc17 and Pt9, the annealing and extension time were extended to avoid large allele dropout:

Step	Initial denaturation	Denaturation	Annealing	Primer extension	final extension
Time (min)	5:00	0:30	**1:45**	**0:45**	15:00
T (°C)	95	95	T_a	72	68
		35 cycles			

References

Cheng WJ, Liu TT, Wu HL, Zhou SB, Xuan SQ, Zhu GP (2010) Isolation and characterization of twelve polymorphic microsatellite loci in Polygonatum cyrtonema and cross-species amplification. Conserv Genet Resour 2:105-107. https://doi.org/10.1007/s12686-010-9218-1

Liu TT, Cheng WJ, Zhou SB, Shao JW, Wu HL, Zhu GP (2010) Eleven polymorphic microsatellite loci in Polygonatum filipes and cross-amplification in other congeneric species. Conserv Genet Resour 2:77-79. https://doi.org/10.1007/s12686-010-9179-4

Sun MZ, Yin X, Shi FX et al (2012) Development of eighteen microsatellite markers in Anemone amurensis (Ranunculaceae) and cross-amplification in congeneric species. Int J Mol Sci 13(4):4889-4895. https://doi.org/10.3390/ijms13044889

S4 Crop dominance

For all land-use parcels mapped as arable field, we determined the dominance of three different crop types over the preceding decade (2008 – 2017): oilseed rape, maize and other cereals. This distinction was based on land-use data generated within the European Integrated Administration and Control System (IACS) (European Commission 2020) and made available by the respective co-authors in each region. These data provide vector geometries of all agricultural land-use parcels and information on the grown crop types for each year. To aggregate the data across years, we converted them to aligned raster data with a cell size of 10 m. For each cell and crop type, we calculated a dominance value between 0 (crop type present in none of the years) and 1 (crop type present in each year). In some regions, data for the years 2008 (West Germany, Central Sweden) and 2009 (Estonia) were not available. Also, some datasets did not cover all arable fields. In general, a dominance value was calculated for a cell, when ≥ 6 layers were available for that cell. To calculate the area of a crop type in a given buffer zone or landscape strip, we multiplied the dominance value of each cell with its cell size (10×10 m²). The area of raster cells, for which no dominance value could be calculated, were subtracted from the total buffer zone or landscape strip area in order to not bias the calculation of percent cover values.

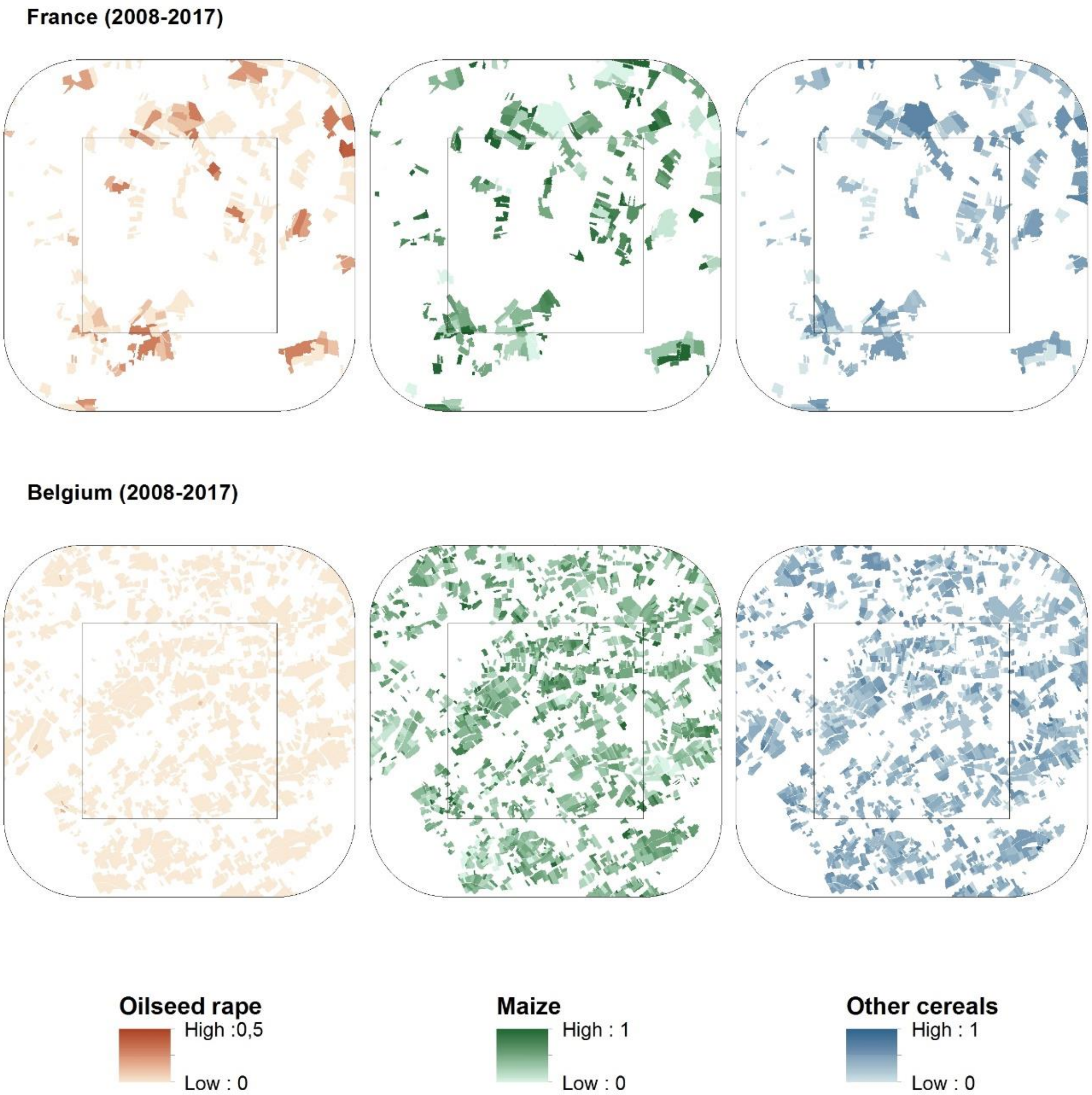

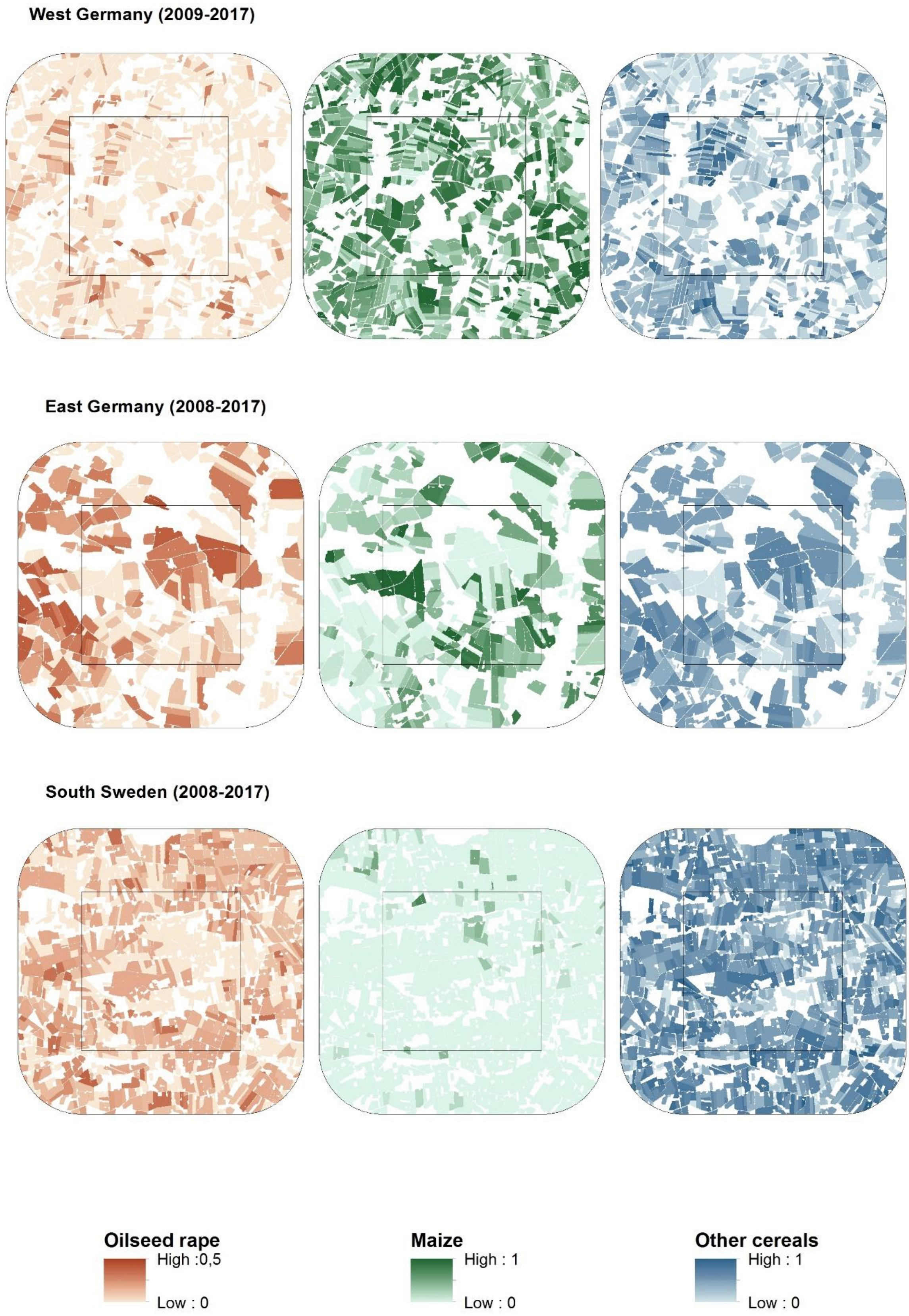
West Germany (2009-2017)
East Germany (2008-2017)
South Sweden (2008-2017)
Oilseed rape
High :0,5
Low : 0
Maize
High : 1
Low : 0
Other cereals
High : 1
Low : 0

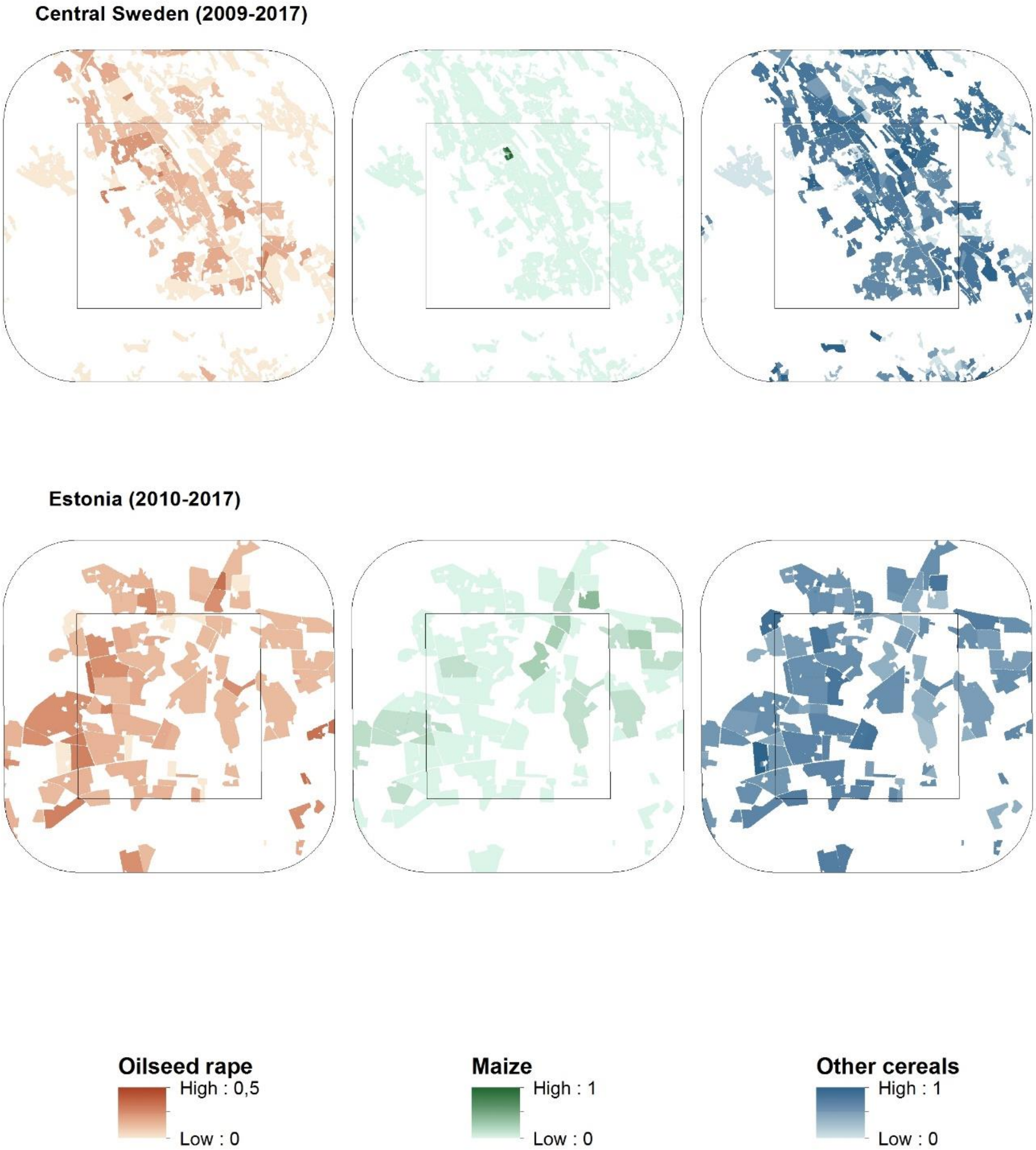
Central Sweden (2009-2017)
Estonia (2010-2017)
Oilseed rape
High : 0,5
Low : 0
Maize
High : 1
Low : 0
Other cereals
High : 1
Low : 0

S5 Additional information on the expected landscape effects shown in Table 1 in the main text

Table S5.1 References for Table 1 in the main text

1 Ahmed KSD, Volpato A, Day MF et al (2021) Linear habitats across a range of farming intensities contribute differently to dipteran abundance and diversity. Insect Conservation and Diversity 14(3):335-347. https://doi.org/10.1111/icad.12455

2 Amici A, Serrani F, Rossi CM, Primi R (2012) Increase in crop damage caused by wild boar (Sus scrofa L.): the "refuge effect". Agron Sustain Dev 32(3):683-692. https://doi.org/10.1007/s13593-011-0057-6

3 Bartual AM, Sutter L, Bocci G et al (2019) The potential of different semi-natural habitats to sustain pollinators and natural enemies in European agricultural landscapes. Agric Ecosyst Environ 279:43-52. https://doi.org/10.1016/j.agee.2019.04.009

4 Batary P, Kovacs-Hostyanszki A, Fischer C, Tscharntke T, Holzschuh A (2012) Contrasting effect of isolation of hedges from forests on farmland vs. woodland birds. Community Ecology 13(2):155-161. https://doi.org/10.1556/ComEc.13.2012.2.4

5 Bennett AF, Hinsley SA, Bellamy PE, Swetnam RD, Mac Nally R (2004) Do regional gradients in land-use influence richness, composition and turnover of bird assemblages in small woods? Biol Conserv 119(2):191-206. https://doi.org/10.1016/j.biocon.2003.11.003

6 Bhattacharya M, Primack RB, Gerwein J (2003) Are roads and railroads barriers to bumblebee movement in a temperate suburban conservation area? Biol Conserv 109(1):37-45. https://doi.org/10.1016/s0006-3207(02)00130-1

7 Bommarco R, Marini L, Vaissiere BE (2012) Insect pollination enhances seed yield, quality, and market value in oilseed rape. Oecologia 169(4):1025-1032. https://doi.org/10.1007/s00442-012-2271-6

8 Bonnot N, Morellet N, Verheyden H et al (2013) Habitat use under predation risk: hunting, roads and human dwellings influence the spatial behaviour of roe deer. Eur J Wildl Res 59(2):185-193. https://doi.org/10.1007/s10344-012-0665-8

9 Breitbach N, Boehning-Gaese K, Laube I, Schleuning M (2012) Short seed-dispersal distances and low seedling recruitment in farmland populations of bird-dispersed cherry trees. J Ecol 100(6):1349-1358. https://doi.org/10.1111/1365-2745.12001

10 Breyne P, Mergeay J, Casaer J (2014) Roe deer population structure in a highly fragmented landscape. Eur J Wildl Res 60(6):909-917. https://doi.org/10.1007/s10344-014-0859-3

11 Chateil C, Porcher E (2015) Landscape features are a better correlate of wild plant pollination than agricultural practices in an intensive cropping system. Agric Ecosyst Environ 201:51-57. https://doi.org/10.1016/j.agee.2014.12.008

12 Collett TS, Graham P (2015) Insect navigation: do honeybees learn to follow highways? Current Biology 25(6):R240-R242. https://doi.org/10.1016/j.cub.2014.11.003

13 Coulon A, Morellet N, Goulard M, Cargnelutti B, Angibault J-M, Hewison AJM (2008) Inferring the effects of landscape structure on roe deer (*Capreolus capreolus*) movements using a step selection function. Landsc Ecol 23(5):603-614. https://doi.org/10.1007/s10980-008-9220-0

14 Cranmer L, McCollin D, Ollerton J (2012) Landscape structure influences pollinator movements and directly affects plant reproductive success. Oikos 121(4):562-568. https://doi.org/10.1111/j.1600-0706.2011.19704.x

15 Cussans J, Goulson D, Sanderson R, Goffe L, Darvill B, Osborne JL (2010) Two bee-pollinated plant species show higher seed production when grown in gardens compared to arable farmland. Plos One 5(7):10. https://doi.org/10.1371/journal.pone.0011753

16 Dainese M, Montecchiari S, Sitzia T, Sigura M, Marini L (2017) High cover of hedgerows in the landscape supports multiple ecosystem services in Mediterranean cereal fields. J Appl Ecol 54(2):380-388. https://doi.org/10.1111/1365-2664.12747

17 Diekötter T, Kadoya T, Peter F, Wolters V, Jauker F (2010) Oilseed rape crops distort plant-pollinator interactions. J Appl Ecol 47(1):209-214. https://doi.org/10.1111/j.1365-2664.2009.01759.x
18 Evans DM, Levey DJ, Tewksbury JJ (2013) Landscape corridors promote long-distance seed dispersal by birds during winter but not during summer at an experimentally fragmented restoration site. Ecological Restoration 31(1):23-30. https://doi.org/10.3368/er.31.1.23
19 Fuller RJ, Chamberlain DE, Burton NHK, Gough SJ (2001) Distributions of birds in lowland agricultural landscapes of England and Wales: How distinctive are bird communities of hedgerows and woodland? Agric Ecosyst Environ 84(1):79-92. https://doi.org/10.1016/s0167-8809(00)00194-8
20 Garcia D, Zamora R, Amico GC (2010) Birds as suppliers of seed dispersal in temperate ecosystems: conservation guidelines from real-world landscapes. Conserv Biol 24(4):1070-1079. https://doi.org/10.1111/j.1523-1739.2009.01440.x
21 Garratt MPD, Senapathi D, Coston DJ, Mortimer SR, Potts SG (2017) The benefits of hedgerows for pollinators and natural enemies depends on hedge quality and landscape context. Agric Ecosyst Environ 247:363-370. https://doi.org/10.1016/j.agee.2017.06.048
22 Goulson D, Lepais O, O'Connor S et al (2010) Effects of land use at a landscape scale on bumblebee nest density and survival. J Appl Ecol 47(6):1207-1215. https://doi.org/10.1111/j.1365-2664.2010.01872.x
23 Grünewald C, Breitbach N, Bohning-Gaese K (2010) Tree visitation and seed dispersal of wild cherries by terrestrial mammals along a human land-use gradient. Basic Appl Ecol 11(6):532-541. https://doi.org/10.1016/j.baae.2010.07.007
24 Haas CA (1995) Dispersal and use of corridors by birds in wooded patches on an agricultural landscape. Conserv Biol 9(4):845-854. https://doi.org/10.1046/j.1523-1739.1995.09040845.x
25 Haenke S, Kovacs-Hostyanszki A, Frund J et al (2014) Landscape configuration of crops and hedgerows drives local syrphid fly abundance. J Appl Ecol 51(2):505-513. https://doi.org/10.1111/1365-2664.12221
26 Hanley ME, Franco M, Dean CE et al (2011) Increased bumblebee abundance along the margins of a mass flowering crop: evidence for pollinator spill-over. Oikos 120(11):1618-1624. https://doi.org/10.1111/j.1600-0706.2011.19233.x
27 Hass AL, Brachmann L, Batary P, Clough Y, Behling H, Tscharntke T (2019) Maize-dominated landscapes reduce bumblebee colony growth through pollen diversity loss. J Appl Ecol 56(2):294-304. https://doi.org/10.1111/1365-2664.13296
28 Hass AL, Kormann UG, Tscharntke T et al (2018) Landscape configurational heterogeneity by small-scale agriculture, not crop diversity, maintains pollinators and plant reproduction in western Europe. Proc R Soc B-Biol Sci 285(1872):10. https://doi.org/10.1098/rspb.2017.2242
29 Heikkinen RK, Luoto M, Virkkala R, Rainio K (2004) Effects of habitat cover, landscape structure and spatial variables on the abundance of birds in an agricultural-forest mosaic. J Appl Ecol 41(5):824-835. https://doi.org/10.1111/j.0021-8901.2004.00938.x
30 Herrault PA, Larrieu L, Cordier S et al (2016) Combined effects of area, connectivity, history and structural heterogeneity of woodlands on the species richness of hoverflies (Diptera: Syrphidae). Landsc Ecol 31(4):877-893. https://doi.org/10.1007/s10980-015-0304-3
31 Herrera JM, Teixeira IdS, Rodriguez-Perez J, Mira A (2016) Landscape structure shapes carnivore-mediated seed dispersal kernels. Landsc Ecol 31(4):731-743. https://doi.org/10.1007/s10980-015-0283-4
32 Herrero J, Garcia-Serrano A, Couto S, Ortuno VM, Garcia-Gonzalez R (2006) Diet of wild boar *Sus scrofa* L. and crop damage in an intensive agroecosystem. Eur J Wildl Res 52(4):245-250. https://doi.org/10.1007/s10344-006-0045-3
33 Herrmann F, Westphal C, Moritz RFA, Steffan-Dewenter I (2007) Genetic diversity and mass resources promote colony size and forager densities of a social bee (*Bombus pascuorum*) in agricultural landscapes. Mol Ecol 16(6):1167-1178. https://doi.org/10.1111/j.1365-294X.2007.03226.x

34 Hinsley SA, Bellamy PE, Newton I, Sparks TH (1995) Habitat and landscape factors influencing the presence of individual breeding bird species in woodland fragments. Journal of Avian Biology 26(2):94-104. https://doi.org/10.2307/3677057
35 Holzschuh A, Dainese M, Gonzalez-Varo JP et al (2016) Mass-flowering crops dilute pollinator abundance in agricultural landscapes across Europe. Ecol Lett 19(10):1228-1236. https://doi.org/10.1111/ele.12657
36 Holzschuh A, Dormann CF, Tscharntke T, Steffan-Dewenter I (2011) Expansion of mass-flowering crops leads to transient pollinator dilution and reduced wild plant pollination. Proc R Soc B-Biol Sci 278(1723):3444-3451. https://doi.org/10.1098/rspb.2011.0268
37 Hudewenz A, Klein AM, Scherber C et al (2012) Herbivore and pollinator responses to grassland management intensity along experimental changes in plant species richness. Biol Conserv 150(1):42-52. https://doi.org/10.1016/j.biocon.2012.02.024
38 Ikin K, Barton PS, Stirnemann IA et al (2014) Multi-scale associations between vegetation cover and woodland bird communities across a large agricultural region. Plos One 9(5). https://doi.org/10.1371/journal.pone.0097029
39 Jakobsson A, Agren J (2014) Distance to semi-natural grassland influences seed production of insect-pollinated herbs. Oecologia 175(1):199-208. https://doi.org/10.1007/s00442-014-2904-z
40 Kamm U, Gugerli F, Rotach P, Edwards P, Holderegger R (2010) Open areas in a landscape enhance pollen-mediated gene flow of a tree species: evidence from northern Switzerland. Landsc Ecol 25(6):903-911. https://doi.org/10.1007/s10980-010-9468-z
41 Keuling O, Stier N, Roth M (2009) Commuting, shifting or remaining? Different spatial utilisation patterns of wild boar *Sus scrofa* L. in forest and field crops during summer. Mamm Biol 74(2):145-152. https://doi.org/10.1016/j.mambio.2008.05.007
42 Klaus F, Bass J, Marholt L, Müller B, Klatt B, Kormann U (2015) Hedgerows have a barrier effect and channel pollinator movement in the agricultural landscape. J Landsc Ecol 8(1):22. https://doi.org/https://doi.org/10.1515/jlecol-2015-0001
43 Knight ME, Osborne JL, Sanderson RA, Hale RJ, Martin AP, Goulson D (2009) Bumblebee nest density and the scale of available forage in arable landscapes. Insect Conservation and Diversity 2(2):116-124. https://doi.org/10.1111/j.1752-4598.2009.00049.x
44 Kovacs-Hostyanszki A, Haenke S, Batary P et al (2013) Contrasting effects of mass-flowering crops on bee pollination of hedge plants at different spatial and temporal scales. Ecol Appl 23(8):1938-1946. https://doi.org/10.1890/12-2012.1
45 Krewenka KM, Holzschuh A, Tscharntke T, Dormann CF (2011) Landscape elements as potential barriers and corridors for bees, wasps and parasitoids. Biol Conserv 144(6):1816-1825. https://doi.org/10.1016/j.biocon.2011.03.014
46 Kreyer D, Oed A, Walther-Hellwig K, Frankl R (2004) Are forests potential landscape barriers for foraging bumblebees? Landscape scale experiments with *Bombus terrestris* agg. and *Bombus pascuorum* (Hymenoptera, Apidae). Biol Conserv 116(1):111-118. https://doi.org/10.1016/s0006-3207(03)00182-4
47 Levey DJ, Bolker BM, Tewksbury JJ, Sargent S, Haddad NM (2005) Effects of landscape corridors on seed dispersal by birds. Science 309(5731):146-148. https://doi.org/10.1126/science.1111479
48 Lövei GL, Macleod A, Hickman JM (1998) Dispersal and effects of barriers on the movement of the New Zealand hover fly *Melanostoma fasciatum* (Dipt., Syrphidae) on cultivated land. J Appl Entomol 122(2-3):115-120.
49 Martin EA, Dainese M, Clough Y et al (2019) The interplay of landscape composition and configuration: new pathways to manage functional biodiversity and agroecosystem services across Europe. Ecol Lett 22(7):1083-1094. https://doi.org/10.1111/ele.13265
50 Meyer B, Jauker F, Steffan-Dewenter I (2009) Contrasting resource-dependent responses of hoverfly richness and density to landscape structure. Basic Appl Ecol 10(2):178-186. https://doi.org/10.1016/j.baae.2008.01.001

51 Mola JM, Miller MR, O'Rourke SM, Williams NM (2020) Forests do not limit bumble bee foraging movements in a montane meadow complex. Ecological Entomology 45(5):955-965. https://doi.org/10.1111/een.12868
52 Montague-Drake RM, Lindenmayer DB, Cunningham RB (2009) Factors affecting site occupancy by woodland bird species of conservation concern. Biol Conserv 142(12):2896-2903. https://doi.org/10.1016/j.biocon.2009.07.009
53 Montero-Castano A, Ortiz-Sanchez FJ, Vila M (2016) Mass flowering crops in a patchy agricultural landscape can reduce bee abundance in adjacent shrublands. Agric Ecosyst Environ 223:22-30. https://doi.org/10.1016/j.agee.2016.02.019
54 Moquet L, Laurent E, Bacchetta R, Jacquemart AL (2018) Conservation of hoverflies (Diptera, Syrphidae) requires complementary resources at the landscape and local scales. Insect Conservation and Diversity 11(1):72-87. https://doi.org/10.1111/icad.12245
55 Morelle K, Lejeune P (2015) Seasonal variations of wild boar *Sus scrofa* distribution in agricultural landscapes: a species distribution modelling approach. Eur J Wildl Res 61(1):45-56. https://doi.org/10.1007/s10344-014-0872-6
56 Morellet N, Van Moorter B, Cargnelutti B et al (2011) Landscape composition influences roe deer habitat selection at both home range and landscape scales. Landsc Ecol 26(7):999-1010. https://doi.org/10.1007/s10980-011-9624-0
57 Nakamura S, Kudo G (2019) The influence of garden flowers on pollinator visits to forest flowers: comparison of bumblebee habitat use between urban and natural areas. Urban Ecosystems 22(6):1097-1112. https://doi.org/10.1007/s11252-019-00891-5
58 Nasiadka P, Janiszewski P (2015) Food preferences of wild boars (Sus scrofa L.) in the summer and early autumn expressed by the damage caused in agricultural crops. Sylwan 159(4):307-317.
59 Pfister SC, Sutter L, Albrecht M, Marini S, Schirmel J, Entling MH (2017) Positive effects of local and landscape features on predatory flies in European agricultural landscapes. Agric Ecosyst Environ 239:283-292. https://doi.org/10.1016/j.agee.2017.01.032
60 Proesmans W, Bonte D, Smagghe G, Meeus I, Verheyen K (2019) Importance of forest fragments as pollinator habitat varies with season and guild. Basic Appl Ecol 34:95-107. https://doi.org/10.1016/j.baae.2018.08.004
61 Proesmans W, Smagghe G, Meeus I, Bonte D, Verheyen K (2019) The effect of mass-flowering orchards and semi-natural habitat on bumblebee colony performance. Landsc Ecol 34(5):1033-1044. https://doi.org/10.1007/s10980-019-00836-5
62 Quinn ACD, Williams DM, Porter WF (2013) Landscape structure influences space use by white-tailed deer. Journal of Mammalogy 94(2):398-407. https://doi.org/10.1644/11-mamm-a-221.1
63 Radford JQ, Bennett AF (2007) The relative importance of landscape properties for woodland birds in agricultural environments. J Appl Ecol 44(4):737-747. https://doi.org/10.1111/j.1365-2664.2007.01327.x
64 Redhead JW, Dreier S, Bourke AFG et al (2016) Effects of habitat composition and landscape structure on worker foraging distances of five bumble bee species. Ecol Appl 26(3):726-739. https://doi.org/10.1890/15-0546
65 Riedinger V, Mitesser O, Hovestadt T, Steffan-Dewenter I, Holzschuh A (2015) Annual dynamics of wild bee densities: attractiveness and productivity effects of oilseed rape. Ecology 96(5):1351-1360. https://doi.org/10.1890/14-1124.1
66 Rosanigo MP, Marrero HJ, Torretta JP (2020) Limiting resources on the reproductive success of a cavity-nesting bee species in a grassland agroecosystem. J Apicult Res 59(4):583-591. https://doi.org/10.1080/00218839.2020.1726034
67 Ruedisser J, Walde J, Tasser E, Fruehauf J, Teufelbauer N, Tappeiner U (2015) Biodiversity in cultural landscapes: influence of land use intensity on bird assemblages. Landsc Ecol 30(10):1851-1863. https://doi.org/10.1007/s10980-015-0215-3
68 Saab V (1999) Importance of spatial scale to habitat use by breeding birds in riparian forests: A hierarchical analysis. Ecol Appl 9(1):135-151. https://doi.org/10.2307/2641174

69 Saïd S, Servanty S (2005) The influence of landscape structure on female roe deer home-range size. Landsc Ecol 20(8):1003-1012. https://doi.org/10.1007/s10980-005-7518-8
70 Schirmel J, Albrecht M, Bauer PM, Sutter L, Pfister SC, Entling MH (2018) Landscape complexity promotes hoverflies across different types of semi-natural habitats in farmland. J Appl Ecol 55(4):1747-1758. https://doi.org/10.1111/1365-2664.13095
71 Schley L, Dufrene M, Krier A, Frantz AC (2008) Patterns of crop damage by wild boar (*Sus scrofa*) in Luxembourg over a 10-year period. Eur J Wildl Res 54(4):589-599. https://doi.org/10.1007/s10344-008-0183-x
72 Sirami C, Gross N, Baillod AB et al (2019) Increasing crop heterogeneity enhances multitrophic diversity across agricultural regions. Proc Natl Acad Sci USA 116(33):16442-16447. https://doi.org/10.1073/pnas.1906419116
73 Steffan-Dewenter I, Munzenberg U, Burger C, Thies C, Tscharntke T (2002) Scale-dependent effects of landscape context on three pollinator guilds. Ecology 83(5):1421-1432. https://doi.org/10.2307/3071954
74 Tenhumberg B, Poehling HM (1995) Syrphids as natural enemies of cereal aphids in Germany - aspects of their biology and efficacy in different years and regions. Agric Ecosyst Environ 52(1):39-43. https://doi.org/10.1016/0167-8809(94)09007-t
75 Thurfjell H, Ball JP, Ahlen PA, Kornacher P, Dettki H, Sjoberg K (2009) Habitat use and spatial patterns of wild boar *Sus scrofa* (L.): agricultural fields and edges. Eur J Wildl Res 55(5):517-523. https://doi.org/10.1007/s10344-009-0268-1
76 Tillmann JE (2011) Bewertung von Maisäckern als Lebensraum für die Tierwelt der Agrarlandschaft mit Hilfe von Fotofallen. Umw Raum 2:43-58.
77 Trombulak SC, Frissell CA (2000) Review of ecological effects of roads on terrestrial and aquatic communities. Conserv Biol 14(1):18-30. https://doi.org/10.1046/j.1523-1739.2000.99084.x
78 Trzcinski MK, Fahrig L, Merriam G (1999) Independent effects of forest cover and fragmentation on the distribution of forest breeding birds. Ecol Appl 9(2):586-593.
79 Van Reeth C, Michel N, Bockstaller C, Caro G (2019) Influences of oilseed rape area and aggregation on pollinator abundance and reproductive success of a co-flowering wild plant. Agric Ecosyst Environ 280:35-42. https://doi.org/10.1016/j.agee.2019.04.025
80 Vray S, Rollin O, Rasmont P, Dufrêne M, Michez D, Dendoncker N (2019) A century of local changes in bumblebee communities and landscape composition in Belgium. J Insect Conserv 23(3):489-501. https://doi.org/10.1007/s10841-019-00139-9
81 Westerbergh A, Saura A (1994) Gene flow and pollinator behavior in *Silene dioica* populations. Oikos 71(2):215-224. https://doi.org/10.2307/3546269
82 Westphal C, Steffan-Dewenter I, Tscharntke T (2003) Mass flowering crops enhance pollinator densities at a landscape scale. Ecol Lett 6(11):961-965. https://doi.org/10.1046/j.1461-0248.2003.00523.x
83 Westphal C, Steffan-Dewenter I, Tscharntke T (2009) Mass flowering oilseed rape improves early colony growth but not sexual reproduction of bumblebees. J Appl Ecol 46(1):187-193. https://doi.org/10.1111/j.1365-2664.2008.01580.x
84 Winfree R, Griswold T, Kremen C (2007) Effect of human disturbance on bee communities in a forested ecosystem. Conserv Biol 21(1):213-223. https://doi.org/10.1111/j.1523-1739.2006.00574.x
85 Wratten SD, Bowie MH, Hickman JM, Evans AM, Sedcole JR, Tylianakis JM (2003) Field boundaries as barriers to movement of hover flies (Diptera: Syrphidae) in cultivated land. Oecologia 134(4):605-611. https://doi.org/10.1007/s00442-002-1128-9
86 Zurbuchen A, Bachofen C, Müller A, Hein S, Dorn S (2010) Are landscape structures insurmountable barriers for foraging bees? A mark-recapture study with two solitary pollen specialist species. Apidologie 41(4):497-508. https://doi.org/10.1051/apido/2009084

Table S5.2 Potential mechanisms how landscape composition and configuration might affect gene flow among spatially isolated populations of the three temperate forest herbs *Anemone nemorosa*, *Oxalis acetosella* and *Polygonatum multiflorum* via pollen- or seed-dispersal vectors according to literature (cf. references in Table 1 in the main text). The direction of the corresponding effect is indicated by - (negative), + (positive) or -+ (negative or positive depending on conditions). Effects of limited importance are written in parentheses

**Landscape metric**	**Potential mechanism**			
**Species:**	***Anemone nemorosa*** ***Oxalis acetosella***	***Oxalis acetosella***	***Polygonatum multiflorum***	***Polygonatum multiflorum***
**Vector:**	**hoverflies, bees**	**wild boar or deer species**	**bumblebees**	**birds or mammals**
**Area-based metrics**				
Percent cover of …				
deciduous forest	- high resistance, + foraging habitat, + resting habitat,	+ foraging habitat, + shelter habitat	- high resistance	+ foraging habitat, + nesting habitat, + resting or shelter habitat
grassland in general	(+ foraging habitat), (+ nesting habitat)	+ foraging habitat	(+ foraging habitat), (+ nesting habitat)	(+ foraging habitat)
semi-natural grassland	+ foraging habitat, + nesting habitat, + spillover to other habitats due to high abundance	+ foraging habitat	- dilution of pollinators and/or distraction from forest herb populations, + foraging habitat, + nesting habitat, + spillover to other habitats due to high abundance	+ foraging habitat
other semi-natural vegetation	+ foraging habitat, + nesting habitat, + spillover to other habitats due to high abundance		- dilution of pollinators and/or distraction from forest herb populations, + foraging habitat, + nesting habitat, + spillover to other habitats due to high abundance	+ foraging habitat
arable land in general	- no habitat, (- high resistance), (+ foraging habitat for aphidophagous hoverfly larvae),	(+ foraging habitat)		- no habitat
arable land cultivated with oilseed rape	- dilution of pollinators and/or distraction from forest herb populations, + temporal foraging habitat, + spillover to other habitats due to high abundance		- dilution of pollinators and/or distraction from forest herb populations, + temporal foraging habitat, + spillover to other habitats due to high abundance	- no habitat
arable land cultivated with maize	- no habitat	+ foraging habitat, + shelter habitat	- no habitat	- no habitat
arable land cultivated with other cereals	- no habitat for bees and adult hoverflies, (- high resistance), + foraging habitat for aphidophagous hoverfly larvae,	(+ foraging habitat)		- no habitat
traditional grassland orchards	- dilution of pollinators and/or distraction from forest herb populations, + temporal foraging habitat, + spillover to other habitats due to high abundance		- dilution of pollinators and/or distraction from forest herb populations, + temporal foraging habitat	
settlement area		- avoided land-use type, - no habitat	- dilution of pollinators and/or distraction from forest herb populations, (- no habitat), + foraging habitat, (+ nesting habitat), (+ spillover to other habitats due to high abundance)	- avoided land-use type, - no habitat

Landscape metric	Potential mechanism			
Species:	***Anemone nemorosa Oxalis acetosella***	***Oxalis acetosella***	***Polygonatum multiflorum***	***Polygonatum multiflorum***
Vector:	**hoverflies, bees**	**wild boar or deer species**	**bumblebees**	**birds or mammals**
**Linear landscape elements**				
Relative length [m ha^{-1}] of …				
hedgerows and tree lines	-+ guidance of movements, + foraging habitat, + resting habitat, + spillover to other habitats due to high abundance,	-+ guidance of movements	-+ guidance of movements, + foraging habitat, + nesting habitat	-+ guidance of movements, + foraging habitat, + nesting habitat, + resting or shelter habitat
water courses (incl. draining ditches)	-+ guidance of movements, (+ foraging habitat)	-+ guidance of movements	-+ guidance of movements, + foraging habitat, + nesting habitat	
broad herbaceous fringes	-+ guidance of movements, + foraging habitat, + nesting habitat, + spillover to other habitats due to high abundance	-+ guidance of movements	-+ guidance of movements, + foraging habitat, + nesting habitat, + spillover to other habitats due to high abundance	+ foraging habitat
roads	- avoided land-use type, -+ guidance of movements	avoided land-use type, -+ guidance of movements	-+ guidance of movements	- avoided land-use type, -+ guidance of movements
**Index metrics**				
Shannon diversity of land-use types	+ diversity effect [a]		+ diversity effect [a]	+ diversity effect [a]
Edge density	+- edge density effect [b]	+- edge density effect [b]	+- edge density effect [b]	+- edge density effect [b]

[a] Diversity effect: high diversity of land-use types increases chance to find suitable habitats.

[b] Edge density effect: high density of edges (due to small land-use patch sizes or complex shapes) increases richness and abundance of pollinators and birds (nesting and foraging habitat), but also restricts animal movements across the landscape (barrier effect)

S6 Complete modelling results

Table S6.1 Effects of landscape metrics on genetic diversity variables as resulting from linear mixed models (LMM) at the node level. Besides landscape metrics, the basic population genetic determinants population size (PopSize) and connectivity were included as fixed effects in all models (see main text). Results refer either to an average model (resulting from full averaging across all candidate models with a $\Delta AIC_c < 2$ or to the single best model (if all other candidate models had a $\Delta AIC_c \geq 2$. Given are the (averaged) standardized regression coefficient (Estimate), the (averaged) standard error (Std. Error), the degrees of freedom (DF; only in single best models), either the *z*-value (average models) or the *t*-value (single best models), the *p*-value of the test against zero, the importance value and the number of candidate models in which the terms were included (#Models). See Table 1 in the main text for the definition of landscape metrics

Anemone nemorosa

(a) A_r ~ PopSize + Connectivity + landscape metrics
Number of observations: 42
Average model over all candidate models with $\Delta AIC_C \leq 2$

	Estimate	Std. Error	*z*-value	*p*-value	Importance	#Models
(Intercept)	0.209	0.214	0.965	0.335	NA	NA
PopSize	0.247	0.097	2.463	0.014	1.00	6
Connectivity	0.381	0.113	3.229	0.001	1.00	6
ARABLE_2000	0.575	0.267	2.131	0.033	1.00	6
LFRINGE_500	-0.157	0.115	1.329	0.184	0.78	4
LWOOD_2000	0.166	0.174	0.950	0.342	0.52	3
LWOOD_2000^2	-0.192	0.207	0.922	0.357	0.52	3
GRASS_1000	0.238	0.268	0.882	0.378	0.48	3
LROAD_125	0.040	0.077	0.514	0.607	0.29	2
GRASS_1000^2	-0.022	0.068	0.322	0.747	0.13	1

(b) H_e ~ PopSize + Connectivity + landscape metrics
Number of observations: 42
Average model over all candidate models with $\Delta AIC_C \leq 2$

	Estimate	Std. Error	*z*-value	*p*-value	Importance	#Models
(Intercept)	-0.032	0.212	0.149	0.882	NA	NA
PopSize	0.135	0.149	0.877	0.380	1.00	12
Connectivity	0.701	0.210	3.211	0.001	1.00	12
MAIZE_1000	0.551	0.218	2.426	0.015	1.00	12
pcSETTLE_500 *	0.495	0.220	2.190	0.029	0.95	11
FOREST_2000	-0.104	0.159	0.644	0.520	0.42	6
SEMNATGRASS_500	0.091	0.145	0.613	0.540	0.41	4
SEMNATVEG_125	-0.031	0.096	0.321	0.748	0.18	3
SEMNATGRASS_500^2	0.043	0.109	0.392	0.695	0.17	1
ORCHARD_1000	0.042	0.129	0.323	0.747	0.12	2
SHANNON_500	0.023	0.088	0.256	0.798	0.10	2
LWOOD_2000^2	-0.010	0.061	0.161	0.872	0.04	1
LWOOD_2000	0.005	0.045	0.119	0.905	0.04	1

* Principal component from SETTLE_500 (r>0), LROAD_500 (r>0) and EDGEDEN_500 (r<0)

(c) H_o ~ PopSize + Connectivity + landscape metrics
Number of observations: 39
Average model over all candidate models with $\Delta AIC_C \leq 2$

	Estimate	Std. Error	*z*-value	*p*-value	Importance	#Models
(Intercept)	-0.525	0.248	2.065	0.039	NA	NA
PopSize	0.111	0.119	0.894	0.372	1.00	6
Connectivity	0.030	0.180	0.160	0.873	1.00	6
pcSETTLE_250 *	0.448	0.146	2.967	0.003	1.00	6
pcSETTLE_250^2	0.485	0.158	2.940	0.003	1.00	6
FOREST_2000	-0.209	0.158	1.300	0.193	0.74	4
CEREAL_250	-0.093	0.154	0.602	0.547	0.31	1
GRASS_2000	0.107	0.189	0.562	0.574	0.28	2
RAPE_2000	-0.052	0._125	0.413	0.680	0.17	1
ORCHARD_2000	-0.001	0.043	0.024	0.981	0.13	1
ORCHARD_2000^2	0.054	0.146	0.369	0.712	0.13	1
MAIZE_250	0.040	0.118	0.338	0.735	0.13	1
SHANNON_125	-0.035	0.107	0.320	0.749	0.11	1

* Principal component from SETTLE_250 (*r*>0) and LROAD_250 (*r*>0)

(d) *F* ~ PopSize + Connectivity + landscape metrics
Number of observations: 37
Average model over all candidate models with $\Delta AIC_C \leq 2$

	Estimate	Std. Error	*z*-value	*p*-value	Importance	#Models
(Intercept)	0.486	0.275	1.717	0.086	NA	NA
PopSize	-0.106	0.141	0.716	0.474	1.00	7
Connectivity	0.036	0.192	0.183	0.855	1.00	7
LROAD_250	-0.477	0.149	3.089	0.002	1.00	7
RAPE_500	0.333	0.245	1.339	0.181	0.76	5
RAPE_500^2	-0.219	0.239	0.905	0.365	0.55	3
LROAD_250^2	-0.228	0.268	0.839	0.402	0.51	4
LFRINGE_500	-0.070	0.148	0.470	0.639	0.23	1
CEREAL_125	0.033	0.102	0.323	0.747	0.12	1
ORCHARD_2000	0.009	0.054	0.152	0.879	0.12	1
ORCHARD_2000^2	-0.045	0.131	0.340	0.734	0.12	1
O:P_LROAD_250	0.026	0.092	0.284	0.777	0.09	1
O:P_LROAD : LROAD_250	-0.031	0.117	0.260	0.795	0.09	1

Oxalis acetosella

(e) A_r ~ PopSize + Connectivity + landscape metrics
Number of observations: 30
Average model over all candidate models with $\Delta AIC_C \leq 2$

	Estimate	Std. Error	*z*-value	*p*-value	Importance	#Models
(Intercept)	0.220	0.437	0.474	0.636	NA	NA
PopSize	0.647	0.132	4.700	0.000	1.00	10
Connectivity	0.025	0.295	0.081	0.936	1.00	10
LROAD_500	0.126	0.122	1.001	0.317	0.70	7
LROAD_500^2	-0.311	0.258	1.196	0.232	0.62	6
LWATER_125	0.102	0.128	0.780	0.435	0.47	4
SETTLE_250	-0.117	0.147	0.785	0.432	0.46	5
MAIZE_250	-0.179	0._250	0.708	0.479	0.38	4
MAIZE_250^2	-0.127	0.182	0.686	0.493	0.38	4
SEMNATVEG_500	0.040	0.092	0.425	0.671	0.21	2
ORCHARD_2000	0.019	0.072	0.266	0.790	0.09	1
SHANNON_2000	-0.012	0.051	0.237	0.813	0.08	1
EDGEDEN_500	-0.003	0.039	0.078	0.938	0.08	1
EDGEDEN_500^2	0.018	0.065	0.278	0.781	0.08	1

(f) H_e ~ PopSize + Connectivity + landscape metrics
Number of observations: 30
Single best model of all candidate models (all other candidate models had a $\Delta AIC_C > 2$

	Estimate	Std. Error	DF	*t*-value	*p*-value
(Intercept)	0.615	0.301	18	2.043	0.056
PopSize	0.613	0.079	18	7.755	0.000
Connectivity	-0.084	0.132	18	-0.640	0.531
MAIZE_250	-0.156	0.111	18	-1.403	0.178
MAIZE_250^2	-0.501	0.099	18	-5.040	0.000
SEMNATVEG_500	0.243	0.082	18	2.958	0.008
SEMNATVEG_500^2	-0.223	0.067	18	-3.326	0.004

(g) H_o ~ PopSize + Connectivity + landscape metrics
Number of observations: 34
Single best model of all candidate models (all other candidate models had a $\Delta AIC_C > 2$

	Estimate	Std. Error	DF	*t*-value	*p*-value
(Intercept)	-0.335	0.288	22	-1.163	0.257
PopSize	0.527	0.107	22	4.940	0.000
Connectivity	-0.219	0.169	22	-1.299	0.207
SEMNATGRASS_1000	0.068	0.119	22	0.569	0.575
SEMNATGRASS_1000^2	0.324	0.090	22	3.609	0.002
SEMNATVEG_500	0.399	0.101	22	3.954	0.001
EDGEDEN_125	-0.304	0.088	22	-3.459	0.002

(h) *F* ~ PopSize + Connectivity + landscape metrics
Number of observations: 34
Single best model of all candidate models (all other candidate models had a $\Delta AIC_C > 2$

	Estimate	Std. Error	DF	*t*-value	*p*-value
(Intercept)	0.585	0.216	22	2.714	0.013
PopSize	-0.020	0.156	22	-0.129	0.898
Connectivity	1.082	0.309	22	3.501	0.002
MAIZE_1000	0.270	0.166	22	1.631	0.117
MAIZE_1000^2	-0.603	0.176	22	-3.417	0.002
pcLWOODGRASS_1000 *	0.741	0.303	22	2.447	0.023
LFRINGE_2000	-0.429	0.138	22	-3.107	0.005

* Principal component from GRASS_1000 (r>0) and LWOOD_1000 (r>0)

Polygonatum multiflorum

(i) A_r ~ PopSize + Connectivity + landscape metrics
Number of observations: 36
Single best model over all candidate models (all other candidate models had a $\Delta AIC_C > 2$

	Estimate	Std. Error	DF	*t*-value	*p*-value
(Intercept)	0.000	0.084	24	0.000	1.000
PopSize	0.704	0.115	24	6.116	0.000
Connectivity	0.056	0.107	24	0.518	0.609
LWATER_2000	0.313	0.098	24	3.206	0.004
pcARABvsGRASS_2000 *	-0.653	0.101	24	-6.475	0.000
SETTLE_1000	-0.311	0.124	24	-2.505	0.019
SHANNON_250	0.634	0.119	24	5.327	0.000

* Principal component from GRASS_2000 (r<0), CEREAL_2000 (r>0) and RAPE_2000 (r>0)

(j) H_e ~ PopSize + Connectivity + landscape metrics
Number of observations: 36
Average model over all candidate models with $\Delta AIC_C \leq 2$

	Estimate	Std. Error	*z*-value	*p*-value	Importance	#Models
(Intercept)	0.208	0.552	0.373	0.709	NA	NA
PopSize	0.030	0.219	0.131	0.896	1.00	5
Connectivity	0.420	0.192	2.119	0.034	1.00	5
pcARABvsGRASS_2000 *	-0.311	0.192	1.575	0.115	0.85	4
SEMNATGRASS_250	0.139	0.168	0.798	0.425	0.70	3
SEMNATGRASS_250^2	-0.426	0.326	1.291	0.197	0.70	3
SETTLE_1000	-0.277	0.272	1.003	0.316	0.62	3
SEMNATVEG_2000	-0.019	0.091	0.203	0.839	0.23	2
SEMNATVEG_2000^2	0.080	0.162	0.490	0.624	0.23	2
LROAD_250	-0.037	0.108	0.330	0.741	0.22	1
LROAD_250^2	0.133	0.271	0.486	0.627	0.22	1

* Principal component from GRASS_2000 (r<0), CEREAL_2000 (r>0) and RAPE_2000 (r>0)

(k) H_o ~ PopSize + Connectivity + landscape metrics
Number of observations: 36
Average model over all candidate models with $\Delta AIC_C \leq 2$

	Estimate	Std. Error	*z*-value	*p*-value	Importance	#Models
(Intercept)	-0.013	0.321	0.039	0.969	NA	NA
PopSize	-0.274	0.163	1.631	0.103	1.00	4
Connectivity	0.309	0.157	1.892	0.059	1.00	4
pcSETTLE_1000 *	-0.409	0.287	1.409	0.159	0.75	3
LWATER_1000	0.080	0.139	0.558	0.577	0.53	2
O:P_LWATER	-0.205	0.221	0.917	0.359	0.53	2
O:P_LWATER : LWATER_1000	-0.245	0.256	0.951	0.342	0.53	2
FOREST_2000	-0.142	0.209	0.676	0.499	0.41	2

* Principal component from SETTLE_1000 (r>0) and LROAD_1000 (r>0)

(l) F ~ PopSize + Connectivity + landscape metrics
Number of observations: 36
Average model over all candidate models with $\Delta AIC_C \leq 2$

	Estimate	Std. Error	*z*-value	*p*-value	Importance	#Models
(Intercept)	-0.051	0.270	0.184	0.854	NA	NA
PopSize	0.368	0.144	2.429	0.015	1.00	6
Connectivity	-0.282	0.137	1.959	0.050	1.00	6
FOREST_2000	0.270	0.208	1.270	0.204	0.76	4
SETTLE_250	-0.334	0.281	1.173	0.241	0.67	5
LROAD_2000	0.385	0.309	1.233	0.218	0.67	5
LWOOD_1000	0.086	0.179	0.466	0.641	0.33	1
O:P_LWOOD_1000	0.130	0.226	0.563	0.573	0.33	1
O:P_LWOOD : LWOOD_1000	0.165	0.252	0.652	0.514	0.33	1
SETTLE_250^2	0.074	0.174	0.421	0.674	0.21	2
PROPGURB_250	-0.042	0.124	0.335	0.738	0.14	1
SETTLE_250 : PROPGURB	0.039	0.129	0.294	0.769	0.14	1
SEMNATVEG_500	0.024	0.078	0.297	0.767	0.13	1

Table S6.2 Effects of landscape metrics on pairwise genetic differentiation variables as resulting from MLPE models at the link level. Besides landscape metrics, all models include geographic distance (GeoDist) and potentially its interactions with landscape metrics as fixed effects (see main text). Results refer to an average model resulting from full averaging across all candidate models with a $\Delta AIC_c < 2$. Given are the averaged standardized regression coefficient (Estimate), the averaged standard error (Std. Error), the *z*- and *p*-value of the test against zero, the importance value and the number of candidate models in which the terms were included (#Models). See Table 1 in the main text for the definition of landscape metrics

Anemone nemorosa

(a) G''_{ST} ~ Geographic distance*landscape metrics
Number of observations: 103
Average model over all candidate models with $\Delta AIC_C \leq 2$

	Estimate	Std. Error	*z*-value	*p*-value	Importance	#Models
(Intercept)	-0.198	0.298	0.654	0.513	NA	NA
GeoDist	-0.090	0.061	1.449	0.147	1.00	11
pcARABvsGRASS_1:3 *	-0.251	0._125	1.974	0.048	1.00	11
pcARABvsGRASS_1:3^2	0.248	0.083	2.943	0.003	1.00	11
GeoDist : pcARABvsGRASS_1:3	-0.260	0.060	4.276	0.000	1.00	11
LFRINGE_1:3	0.192	0.075	2.540	0.011	1.00	11
FOREST_1:3	0.040	0.069	0.571	0.568	0.54	6
GeoDist : FOREST_1:3	0.034	0.062	0.540	0.589	0.30	3
FOREST_1:3^2	-0.012	0.030	0.382	0.703	0.24	3
SETTLE_1:7	0.014	0.043	0.319	0.750	0.17	2
GeoDist : FOREST_1:3^2	0.006	0.024	0.241	0.810	0.08	1
SHANNON_1:5	0.005	0.029	0.191	0.849	0.08	1
LWATER_2:3	0.004	0.023	0.178	0.859	0.07	1
LWOOD_2:3	0.010	0.054	0.185	0.853	0.07	1

* Principal component from CEREAL_1:3 (*r*>0), RAPE_1:3 (*r*>0) and GRASS_1:3 (*r*<0)

(b) D_{PS} ~ Geographic distance*landscape metrics
Number of observations: 104
Average model over all candidate models with $\Delta AIC_C \leq 2$

	Estimate	Std. Error	*z*-value	*p*-value	Importance	#Models
(Intercept)	-0.174	0.280	0.614	0.539	NA	NA
GeoDist	-0.089	0.073	1.207	0.227	1.00	8
pcARABvsGRASS_1:2 *	-0.333	0.152	2.163	0.031	1.00	8
pcARABvsGRASS_1:2^2	0.284	0.091	3.076	0.002	1.00	8
GeoDist : pcARABvsGRASS_1:2	-0.239	0.060	3.953	0.000	1.00	8
FOREST_1:3	0.045	0.074	0.598	0.550	1.00	8
FOREST_1:3^2	-0.083	0.050	1.627	0.104	1.00	8
LFRINGE_1:3	0.208	0.086	2.404	0.016	1.00	8
GeoDist : FOREST_1:3^2	0.037	0.056	0.658	0.511	0.42	3
LROAD_1:7	0.038	0.063	0.602	0.547	0.40	3
SEMNATVEG_1:5	-0.022	0.055	0.402	0.687	0.21	2
LWOOD_2:3	0.035	0.102	0.340	0.734	0.17	2

* Principal component from CEREAL_1:2 (*r*>0), RAPE_1:2 (*r*>0) and GRASS_1:2 (*r*<0)

Oxalis acetosella

(c) G''_{ST} ~ Geographic distance*landscape metrics
Number of observations: 78
Single best model of all candidate models (all other candidate models had a $\Delta AIC_C > 2$

	Estimate	Std. Error	DF	*t*-value	*p*-value
(Intercept)	0.482	0.468	63	1.031	0.306
GeoDist	0.277	0.094	63	2.967	0.004
FOREST_1:3	0.136	0.067	63	2.047	0.045
GeoDist : FOREST_1:3	0.193	0.059	63	3.273	0.002
RAPE_1:2	0.203	0._125	63	1.629	0.108
RAPE_1:2^2	-0.230	0.072	63	-3.189	0.002
GeoDist : RAPE_1:2	0.056	0.054	63	1.032	0.306
GeoDist : RAPE_1:2^2	-0.264	0.064	63	-4.144	0.000
LWATER_1:3	-0.218	0.063	63	-3.472	0.001
LROAD_1:5	-0._125	0.060	63	-2.099	0.040

(d) D_{PS} ~ Geographic distance*landscape metrics
Number of observations: 80
Average model over all candidate models with $\Delta AIC_C \leq 2$

	Estimate	Std. Error	*z*-value	*p*-value	Importance	#Models
(Intercept)	0.134	0.337	0.390	0.696	NA	NA
GeoDist	0.082	0.103	0.787	0.432	1.00	3
FOREST_1:3	0.345	0.103	3.305	0.001	1.00	3
LWOOD_1:7	0.270	0.154	1.728	0.084	1.00	3
O:P_LWOOD	-0.070	0.056	1.220	0.222	1.00	3
O:P_LWOOD : LWOOD_1:7	0.216	0.067	3.152	0.002	1.00	3
ARABLE_1:3	0.188	0.143	1.300	0.194	0.80	2
SHANNON_2:3	-0.167	0.138	1.202	0.229	0.70	2
GeoDist : FOREST_1:3	0.100	0.085	1.168	0.243	0.70	2
ARABLE_1:3^2	-0.012	0.044	0.270	0.787	0.30	1
GeoDist : ARABLE_1:3	-0.047	0.082	0.573	0.567	0.30	1
GeoDist : ARABLE_1:3^2	-0.069	0.114	0.608	0.543	0.30	1

Polygonatum multiflorum

(e) G''_{ST} ~ Geographic distance*landscape metrics
Number of observations: 90
Average model over all candidate models with $\Delta AIC_C \leq 2$

	Estimate	Std. Error	*z*-value	*p*-value	Importance	#Models
(Intercept)	-0.294	0.336	0.863	0.388	NA	NA
GeoDist	0.158	0.106	1.469	0.142	1.00	17
SEMNATGRASS_1:3	0.265	0.131	1.992	0.046	1.00	17
SEMNATGRASS_1:3^2	0.267	0.099	2.670	0.008	1.00	17
FOREST_1:2	-0.262	0.160	1.623	0.105	0.87	14
LROAD_1:7	-0.037	0.134	0.275	0.783	0.71	12
GeoDist : FOREST_1:2	-0.101	0.097	1.036	0.300	0.66	10
O:P_LROAD	0.027	0.059	0.455	0.649	0.50	8
O:P_LROAD : LROAD_1:7	0.126	0.144	0.873	0.383	0.50	8
ORCHARD_2:3	0.074	0.118	0.620	0.535	0.41	8
LWATER_2:3	-0.064	0.103	0.611	0.541	0.39	7
ARABLE_1:2	0.000	0.027	0.011	0.992	0.04	1
ARABLE_1:2^2	0.009	0.048	0.192	0.848	0.04	1

(f) D_{PS} ~ Geographic distance*landscape metrics
Number of observations: 87
Average model over all candidate models with $\Delta AIC_C \leq 2$

	Estimate	Std. Error	*z*-value	*p*-value	Importance	#Models
(Intercept)	0.064	0.221	0.285	0.775	NA	NA
GeoDist	0.124	0.133	0.923	0.356	1.00	8
SEMNATGRASS_1:3	0.318	0.131	2.401	0.016	1.00	8
ORCHARD_2:3	0.441	0.160	2.714	0.007	1.00	8
LWOOD_1:5	-0.303	0.141	2.108	0.035	1.00	8
LROAD_1:2	-0.383	0.153	2.463	0.014	1.00	8
O:P_LROAD	0.085	0.110	0.768	0.442	0.44	3
O:P_LROAD : LROAD_1:2	-0.009	0.063	0.140	0.888	0.44	3
MAIZE_1:7	0.062	0.120	0.512	0.609	0.44	4
MAIZE_1:7^2	-0.129	0.171	0.749	0.454	0.44	4
SEMNATGRASS_1:3^2	0.064	0.114	0.560	0.575	0.31	3
LWATER_2:3	-0.023	0.075	0.312	0.755	0.12	1
LFRINGE_1:2	0.013	0.056	0.227	0.821	0.09	1

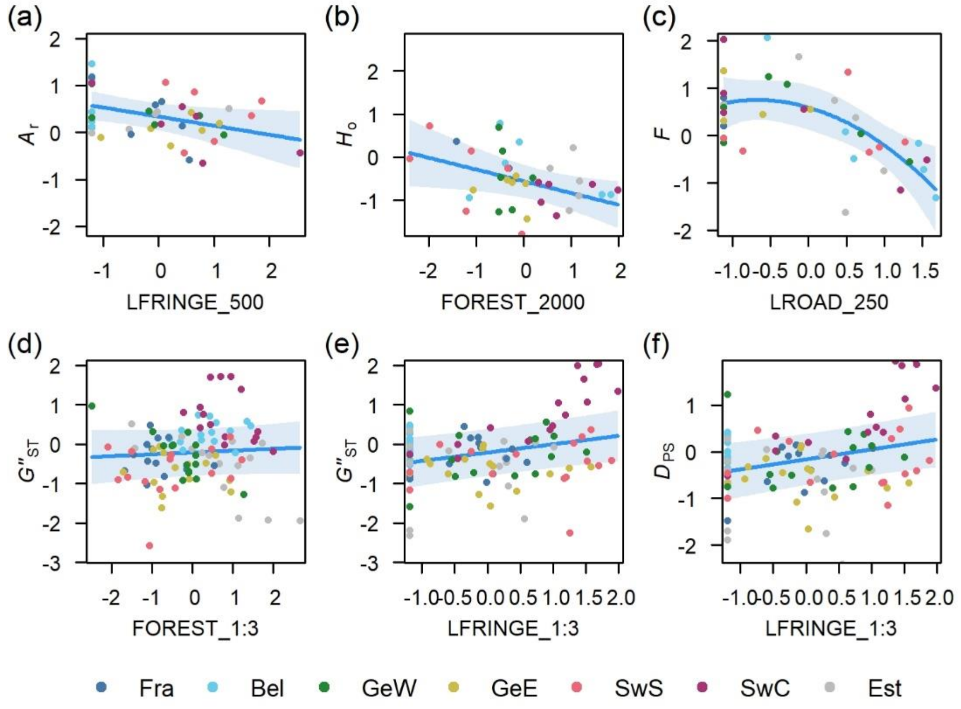

Fig. S6.1 Visualizations of landscape effects on population genetic variables for *Anemone nemorosa* (cf. Tables S6.1 and S6.2, which are not presented in the main text. Shown are the partial slopes and residuals as well as the 95% confidence band. All variables are scaled in standard deviation units. Colors represent the different landscape windows: France (Fra), Belgium (Be), West Germany (GeW), East Germany (GeE), South Sweden (SwS), Central Sweden (SwC), and Estonia (Est). Population genetic variables are allelic richness (A_r), observed heterozygosity (H_o), inbreeding index (F), and genetic differentiation (G''_{ST} and D_{PS}). The landscape metric FOREST refers to the percent cover of deciduous forest. LFRINGE and LROAD refer to the relative length of herbaceous fringes and roads, respectively. Numbers or ratios added to the variable names correspond to the most influential buffer distance in meters or the most influential width-to-length radio of the landscape strips, respectively

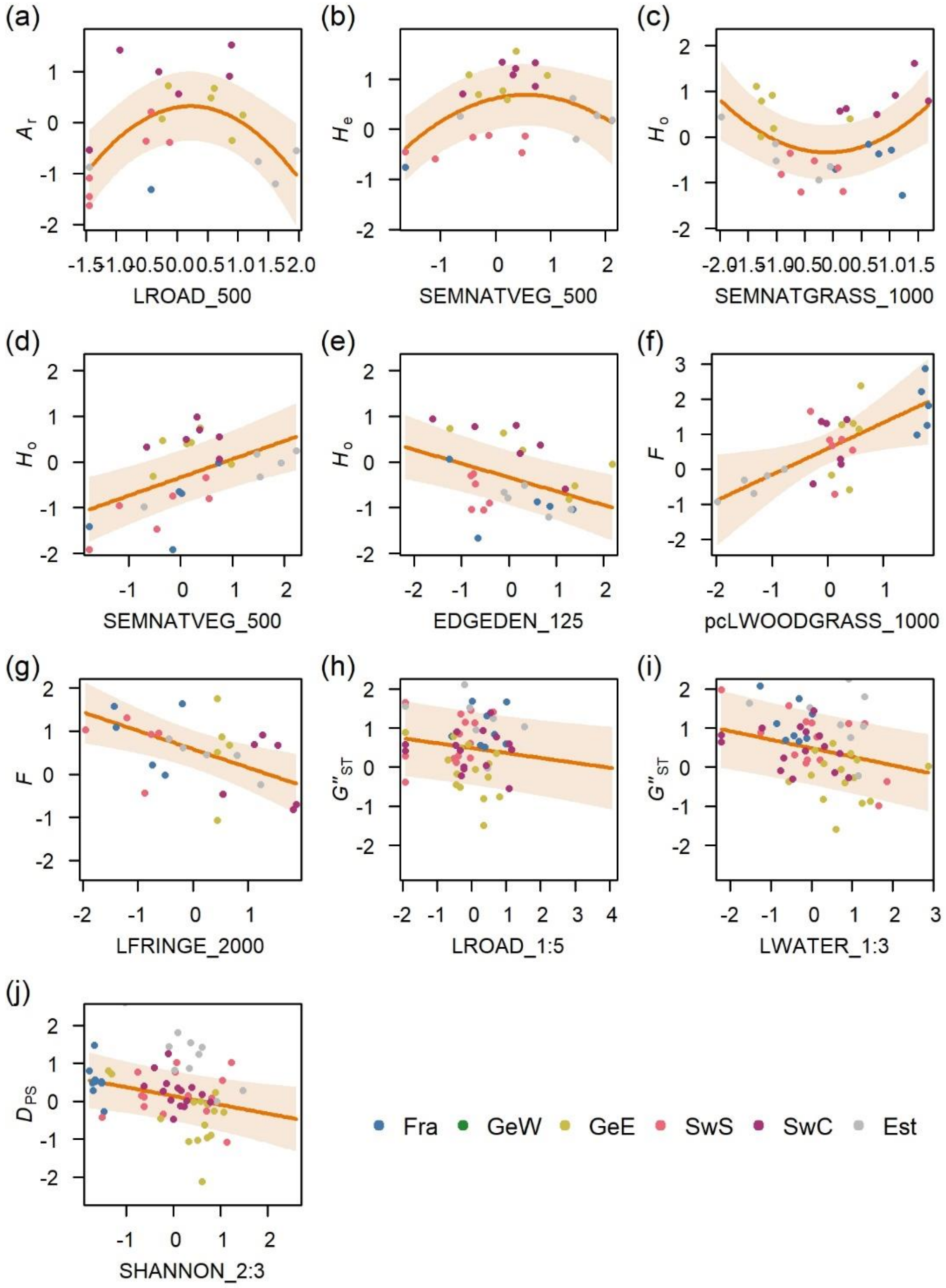

Fig. S6.2 Visualizations of landscape effects on population genetic variables for *Oxalis acetosella* (cf. Tables S6.1 and S6.2, which are not presented in the main text. Shown are the partial slopes and residuals as well as the 95% confidence band. All variables are scaled in standard deviation units. Colors represent the different landscape windows: France (Fra), West Germany (GeW), East Germany (GeE), South Sweden (SwS), Central Sweden (SwC), and Estonia (Est). Population genetic variables are allelic richness (A_r), expected (H_e) and observed heterozygosity (H_o), inbreeding index (F), and genetic differentiation (G''_{ST} and D_{PS}). The landscape metrics SEMNATGRASS and SEMNATVEG refer to the percent cover of semi-natural grassland and other semi-natural vegetation, respectively. LFRINGE, LROAD, and LWATER refer to the relative length of herbaceous fringes, roads and water courses, respectively. EDGEDEN is the land-use parcel edge density. SHANNON is the diversity of land-use types. pcLWOODGRASS is a principal component reflecting the percent cover of grassland and the relative length of hedgerows/treelines (cf. Table 2 in the main text). Numbers or ratios added to the variable names correspond to the most influential buffer distance in meters or the most influential width-to-length radio of the landscape strips, respectively

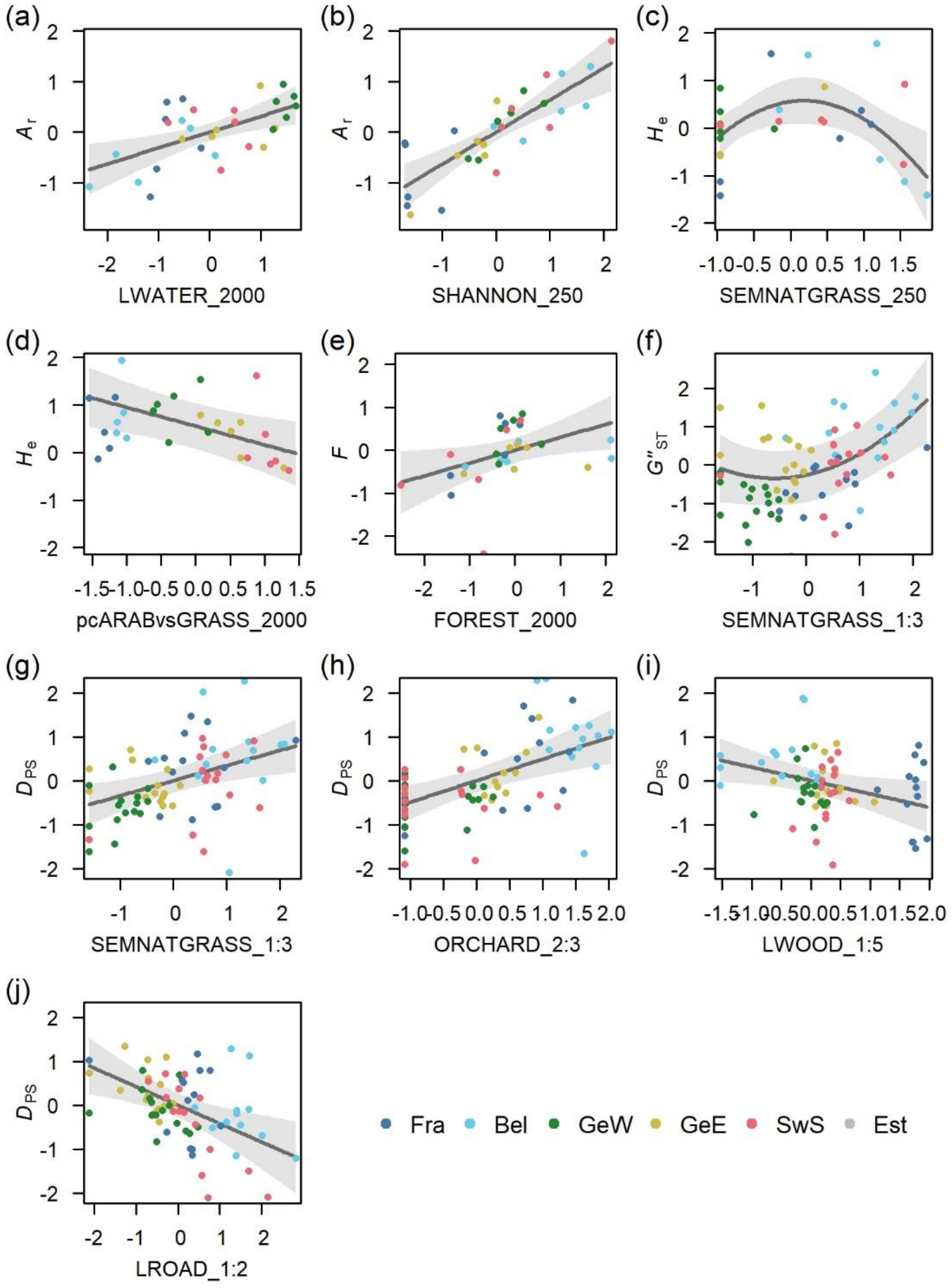

Fig. S7.3 Visualizations of landscape effects on population genetic variables for *Polygonatum multiflorum* (cf. Tables S7.1 and S7.2, which are not presented in the main text. Shown are the partial slopes and residuals as well as the 95% confidence band. All variables are scaled in standard deviation units. Colors represent the different landscape windows: France (Fra), Belgium (Bel), West Germany (GeW), East Germany (GeE), South Sweden (SwS), and Estonia (Est). Population genetic variables are allelic richness (A_r), expected heterozygosity (H_e), inbreeding index (F), and genetic differentiation (G''_{ST} and D_{PS}). The landscape metrics FOREST, ORCHARD, and SEMNATGRASS refer to the percent cover of deciduous forest, traditional grassland orchards, and semi-natural grassland, respectively. LROAD, LWATER, and LWOOD refer to the relative length of roads, water courses, and hedgerows/treelines, respectively. SHANNON is the diversity of land-use types. pcARABvsGRASS is a principal component reflecting the trade-off between arable land (cereals and oilseed rape) on the one hand and grassland on the other hand (cf. Table 2). Numbers or ratios added to the variable names correspond to the most influential buffer distance in meters or the most influential width-to-length radio of the landscape strips, respectively
